# Exploring the impact of polychlorinated biphenyls on comorbidity and potential mitigation strategies

**DOI:** 10.3389/fpubh.2024.1474994

**Published:** 2024-10-30

**Authors:** Ying Gao, Han Lu, Huan Zhou, Jiaxing Tan

**Affiliations:** ^1^Division of Nephrology, Department of Medicine, West China Hospital, Sichuan University, Chengdu, Sichuan, China; ^2^West China School of Medicine, Sichuan University, Chengdu, Sichuan, China; ^3^Computational Mathematics and Machine Learning, School of Mathematics, Sichuan University, Chengdu, Sichuan, China; ^4^Department of Pediatrics, Pennsylvania State University College of Medicine, Hershey, PA, United States

**Keywords:** polychlorinated biphenyls, machine learning, mortality, comorbidities, DII

## Abstract

**Introduction:**

Polychlorinated Biphenyls (PCBs) persist in the environment and accumulate in humans. Currently, there is a lack of understanding about the overall impact of PCBs on human health, and effective interventions for exposed populations are insufficient.

**Methods:**

Our study aimed to assess the impact of PCBs on various diseases and mortality risks using data from the National Health and Nutrition Examination Survey, while proposing lifestyle adjustments, particularly dietary modifications, to mitigate mortality risk. Statistical analyses employed principal component analysis, multifactorial logistic regression, multifactorial Cox regression, comorbidity network analysis, and machine learning prediction models.

**Results:**

Results indicated significant associations between 7 types of PCBs and 12 diseases (*p* < 0.05), with 6 diseases showing significant positive correlations (OR > 1, *p* < 0.05), along with listing the 25 most relevant diseases, such as asthma and chronic bronchitis (OR [95% CI] = 5.85 [4.37, 7.83], *p* < 0.0001), arthritis and osteoporosis (OR [95% CI] = 6.27 [5.23, 7.55], *p* < 0.0001). This suggested that PCBs may be intimately involved in the development and progression of multiple diseases. By constructing multidimensional machine learning models and conducting multiple iterations for precision and error measurement, PCBs may have the potential to become specific biomarkers for certain diseases in the future. Building upon this, we further suggested that controlling dietary intake to reduce dietary inflammatory index (DII) could lower mortality and disease risks.

**Discussion:**

While PCBs were independent risk factors for mortality, substantial evidence suggested that adjusting DII might mitigate the adverse effects of PCBs to some extent. Further physiological mechanisms require deeper exploration through additional research.

## 1 Introduction

Due to the significant adverse effects of PCBs on human health and ecosystems, the United States banned the production of PCBs in 1979 under the Toxic Substances Control Act. However, production continued in other parts of the world. Consequently, the United Nations Environment Program (UNEP) established the Stockholm Convention in May 2001, aiming to eliminate PCB-containing products in an environmentally sound manner by 2028 (1). As part of this effort, the 2001 Stockholm Convention on Persistent Organic Pollutants prohibited the production and use of PCBs—substances once widely used in electrical manufacturing and construction materials due to their lipophilicity, environmental persistence, and bioaccumulation in animal tissues. Despite this, the lingering effects of PCBs in the environment remain a significant concern (2–4). PCBs continue to enter the human body through the food chain and environmental exposure, persisting in various settings (5). Multiple studies have shown that PCBs tend to accumulate significantly in high-fat, high-protein foods that are commonly consumed, such as eggs (6), mussels (7, 8) and dairy products (9). They have also been widely detected in animal feed. Pollution surveys in China have identified significant PCB contamination in electronic waste dismantling areas such as Taizhou City in Zhejiang Province, Qingyuan City and Guiyu Town in Guangdong Province, as well as in major urban and industrial areas, particularly in East China. This contamination largely stems from inadvertent production during industrial heat treatment processes. Furthermore, epidemiological studies indicate detectable levels of PCBs in the human body, underscoring the ongoing potential pollution and hazards associated with these compounds (10, 11).

It should be noted that previous studies indicate that the 209 possible PCB congeners are generally divided into two main categories: “coplanar” and “non-coplanar.” Coplanar PCBs have a more planar molecular structure due to the absence of chlorine substitution at the ortho positions (12). Their molecular planarity resembles that of dioxins, enabling them to bind to the aryl hydrocarbon receptor (AhR) in the body, similarly to dioxin-like substances. This interaction can induce oxidative stress, inflammatory responses, and alterations in gene expression, potentially leading to cancer, immune suppression, and endocrine disruption (13). Dioxin-like PCBs exhibit higher toxicity and may result in chronic toxicity, liver damage, and reproductive and developmental issues (14). In contrast, non-coplanar PCBs have chlorine atoms substituted at the ortho positions, which disrupts their planar structure. This prevents them from binding to the AhR receptor, leading to a different toxicity mechanism compared to coplanar PCBs. The toxicity of non-coplanar PCBs primarily operates through other pathways, such as affecting calcium metabolism and interfering with cell membrane functions (15). They have significant impacts on the nervous system, with studies indicating that these PCBs may influence neurotransmitter transmission, resulting in neurotoxicity, particularly affecting the developing brain and nervous system. Although their toxicity is lower than that of coplanar PCBs, long-term exposure can still lead to cardiovascular diseases, liver issues, and immune system suppression (16).

Previous research has highlighted the potential links between PCB exposure and human health, primarily examining the relationship between PCBs and individual medical conditions such as hypertension, diabetes, pulmonary arterial hypertension, and liver diseases (17). These studies generally focus on the effects of PCBs on specific systems, while neglecting the comprehensive analysis of their impact across multiple biological systems in humans, a complex organism. Moreover, although these studies have identified potential adverse effects of PCBs on human health, solutions for mitigating or preventing these environmental sequelae have been inadequately proposed, even in developed countries where their use has already been banned. This lack of effective strategies leaves public health scholars and clinicians feeling powerless in addressing the ongoing challenges posed by PCB contamination (18–21).

Considering that analyses focusing on individual diseases fail to account for interactions between diseases and that traditional methods for assessing mortality risk do not adequately address issues related to common exposure and risk confounding, comprehensive analyses of PCBs and their impact on comorbidity networks are still lacking (3, 22). To bridge this gap, our study combined traditional statistical analysis with recent machine learning models and utilized data from the National Health and Nutrition Examination Survey (NHANES) to comprehensively investigate the integrated impact of PCB exposure on multiple disease categories and its relationship with mortality. We discovered that PCB exposure contributes to a chronic state of inflammation and disease in the body. For the first time, we propose a strategy to mitigate the adverse effects of PCBs through an anti-inflammatory diet, which can counteract the detrimental consequences of PCBs exposure.

## 2 Materials

### 2.1 Study design and participants

As of March 1, 2024, all data used in our study were publicly accessible and sourced from the NHANES, managed by the National Center for Health Statistics (NCHS). Since 1999, data collection from participants has been conducted through questionnaire interviews, physical examinations, and laboratory tests. NHANES received approval from the NCHS Institutional Review Board, and all participants have provided written informed consent. Survival status data of NHANES participants were derived from the National Death Index (NDI).

Our study selected 69 congeners of persistent organic pollutants detected and validated in the NHANES database (1999–2004), including dioxins, furans, and PCBs. Through data screening and analysis, we identified seven PCBs—PCB074, PCB170, PCB178, PCB180, PCB156, PCB157, and PCB146—as noteworthy compounds. We have provided specific nomenclature and abbreviations for these compounds:

LBX074, representing PCB074(2,4,4′,5-Tetrachlorobiphenyl).

LBX170, representing PCB170(2,2′,3,3′,4,4′,5-Heptachlorobiphenyl).

LBX178, representing PCB178(2,2′,3,3′,5,5′,6-Heptachlorobiphenyl).

LBX180, representing PCB180(2,2′,3,4,4′,5,5′-Heptachlorobiphenyl).

LBX156, representing PCB156(2,3,3′,4,4′,5-Hexachlorobiphenyl).

LBX157, representing PCB157(2,3,3′,4,4′,5′-Hexachlorobiphenyl).

LBX146, representing PCB146(2,3,3′,4,4′,5′-Hexachlorobiphenyl).

It is important to note that these seven PCBs were all non-dioxin-like PCBs. The completeness of the data and the limitations of the detection equipment and methods at the time may have contributed to the underrepresentation of dioxin-like PCBs in the data analysis.

We matched the NHANES data from 1999 to 2004 with mortality data from the NDI website, excluding participants with missing mortality data and incomplete data for the seven PCBs. Additionally, the NHANES database provides a weight for each participant to account for the complex survey design, including oversampling, non-response adjustments, and post-stratification to align with the total census population. When weighted, the sample in NHANES represents the civilian, non-institutionalized population of the United States. Each participant is assigned a sample weight, which reflects the number of individuals represented by that sample. Therefore, we also excluded participants with missing weights. However, for comprehensive network analysis, we refrained from further data exclusion. This meticulous curation resulted in a final cohort of 10,961 participants. At different analysis stages, data were selected based on varying data requirements.

### 2.2 Methodology for measuring serum levels of PCBs

The analyses were quantified in serum using high-resolution gas chromatography and isotope-dilution high-resolution mass spectrometry (HRGS/ID-HRMS). Serum samples were fortified with 13C12-labeled internal standards and extracted through either C18 solid-phase extraction (SPE) or liquid-liquid extraction. Chromatographic separation occurred on a DB-5ms capillary column employing a Hewlett-Packard 6890 gas chromatograph. Quantification was achieved by ID-HRMS using selected ion monitoring (SIM) at a resolving power of 10,000 with either a Micromass AutoSpec ULTIMA or Finnigan MAT95 mass spectrometer in electron ionization (EI) mode. Detection limits were reported for each sample, accounting for sample weight and analyte recovery.

From the entire persistent organic pollutant (POP) library, we ultimately identified 18 PCBs, with data for these substances in the NHANES cycles from 1999–2000, 2001–2002, and 2003–2004 accounting for over 75% of the total data. Further analysis of the 18 POPs using weighted quantile sum (WQS) analysis identified seven PCBs with the greatest impact on mortality risk, cumulatively contributing to 95% of the total weight. As per NHANES guidelines, values falling below the limit of detection (LOD) were imputed with a value equivalent to the LOD divided by the square root of 2.

### 2.3 Diagnosis of medical conditions and DII

As NHANES does not directly record mortality data, mortality information in this study was obtained through a probabilistic match between NHANES and NDI, following procedures validated by the National Center for Health Statistics (NCHS). Mortality data from the NDI were available for analysis until December 31, 2019.

All diseases available in NHANES were included in our analysis, encompassing Angina, Heart attack (HA), Heart disease (HD), Heart failure (HF), Hypertension, Stroke, Alcoholic fatty liver disease (ALD), Hepatitis B virus (HBV), Hepatitis C virus (HCV), Hepatitis D virus (HDV), Non-alcoholic fatty liver disease (NAFLD), Hyperlipidemia, Diabetes, Osteoporosis, Hyperuricemia, Thyroid disease (TD), Human Immunodeficiency Virus (HIV), Arthritis, Depression, Cancer, Chronic bronchitis (CB), Emphysema, Asthma, Chronic kidney disease (CKD), and Proteinuria, totaling 25 diseases. Diabetes was defined as self-reported diabetes diagnosis, use of oral antidiabetic drugs or insulin, glycated hemoglobin (HbA1c) levels ≥6.5%, plasma glucose levels ≥200 mg/dL 2 h after an oral glucose tolerance test (OGTT), or fasting plasma glucose levels ≥126 mg/dL (23). Hypertension was determined based on self-reported hypertension or NHANES-measured data: an average systolic blood pressure ≥130 mm Hg or diastolic blood pressure ≥80 mm Hg from three measurements (24, 25). To assess CKD, essential indicators including estimated glomerular filtration rate (eGFR) and urine albumin-to-creatinine ratio (UACR) were relied upon. UACR (mg/g) was calculated as the ratio of urine albumin (mg/dL) to urine creatinine (g/dL), with a UACR value exceeding 30 mg/g indicating “proteinuria.” eGFR was computed using the CKD-EPI formula, expressed as:

GFR = 175 × standardized serum creatinine^(−1.154)^ × age^(−0.203)^ × 1.212 [if Black] × 0.742 [if female], where serum creatinine is measured in mg/dL.

CKD was defined as eGFR <60 mL/min/1.73 m^2^ the presence of renal damage markers (such as proteinuria), or both, persisting for at least 3 months, regardless of the underlying etiology (26). In line with previous publications, NAFLD was defined by hepatic steatosis index (HSI) and US fatty liver index (USFLI). The formulas are as follows:

HIS = 8 × (alanine aminotransferase/aspartate aminotransferase ratio) + body mass index (+2 for female; +2 for diabetes);

USFLI = (e^−0.8073^ × Non–Hispanic Black+0.3458 × Mexican American+0.0093 × Age+0.6151 × loge (Gamma glutamyltransferase) +0.0249 × Waist Circumference+1.1792 × loge (Insulin)+0.8242 × loge (Glucose)−14.7812)/ (1 + e^−0.8073^ × Non–Hispanic Black+0.3458 × Mexican American+0.0093 × Age+0.6151 × loge (Gamma glutamyltransferase) +0.0249 × waist circumference+1.1792 × loge (Insulin)+0.8242 × loge (Glucose) – 14.7812) × 100.

USFLI cutoff value ≥ 30 or HSI value > 36 was diagnosed as NAFLD (27). ALD was defined by a combination of an evidence of excessive alcohol consumption (≥ 210 g/week for men and ≥ 140 g/week for women) and an ALD/NAFLD index > 0, which was calculated as:

−58.5 + 0.637 (Mean Corpuscular Volume) + 3.91 (Aspartate Aminotransferase [AST]/Alanine Aminotransferase [ALT]) – 0.406 (Body Mass Index) + 6.35 for Male Gender.

In the subpopulation with ALD, the AST-to-platelet ratio index (APRI) and FIB-4 score were used to evaluate ALD FIB. The formula is as follows:

APRI = (AST/Upper Limit of Normal/Platelet Count [109/L]) × 100, where the upper limits of normal AST levels were set at 37 IU/L for men and 29 IU/L for women;

FIB-4 = Age × AST/[Platelets in 109/L × (ALT)^1/2^].

Cut-off values for advanced fibrosis (≥ F3) were set at 1.5 for APRI and 3.25 for FIB-4 (28, 29). HBV, HCV, HDV, and HIV-positive patients were determined based on antigen measurements and quantification of relevant viral DNA or RNA levels in NHANES laboratories. Hyperlipidemia was defined as fasting triglyceride values ≥ 200 ng/dl. Smokers were defined as individuals who have smoked more than 100 cigarettes in their lifetime and currently smoke on some days or every day, while nonsmokers are those who have smoked <100 cigarettes in their lifetime (30). Hyperuricemia was defined dichotomously with serum uric acid (SUA) levels ≥416 μmol/L (7.0 mg/dL) for males and ≥357 μmol/L (6.0 mg/dL) for females (31). Apart from the diseases mentioned above, data on Heart attack, Heart disease, Heart failure, Stroke, Osteoporosis, Thyroid disease, Emphysema, Arthritis, Depression, Cancer, Chronic bronchitis, and Asthma were obtained from questionnaire data provided by NHANES. These indices were derived from comprehensive full blood cell count tests, the details of which can be found in the “Questionnaire” data within the NHANES dataset. Participants who answered “Yes” were considered to have that condition. Finally, we categorized these diseases into seven major classes based on disease type: Circulatory system diseases, Digestive system diseases, Endocrine/Metabolic diseases, Immune system diseases, Respiratory system diseases, Urinary system diseases, and Others.

The DII assesses the inflammatory effect of diet using 45 dietary parameters, normalizing individual intake of each food parameter to global intake. Standardized intake scores (Z-scores) are converted to proportions and centered. The centered proportions of these specific food intakes are multiplied by their inflammation effect scores and summed to obtain an individual's overall DII score. Participants' DII scores represent the sum of each DII score. Higher DII scores indicate a pro-inflammatory diet, while lower scores indicate an anti-inflammatory diet. In this study, 28 out of 45 food parameters were utilized for DII calculation: carbohydrates, protein, total fat, alcohol, fiber, cholesterol, saturated fatty acids, monounsaturated fatty acids, polyunsaturated fatty acids, n-3 fatty acids, n-6 fatty acids, niacin, vitamin A, thiamine, vitamin B2, vitamin B6, vitamin B12, vitamin C, vitamin D, vitamin E, iron, magnesium, zinc, selenium, folic acid, carotene, caffeine, and energy (32, 33).

### 2.4 Covariates

We included C-reactive protein (CRP) and the systemic immune-inflammation index (SII) as covariates in our analysis. We selected and utilized data on platelet count (PC), neutrophil count (NC), and lymphocyte count (LC) in the computation, with SII calculated as SII = PC ^*^ (NC/LC) (34).

Other covariates included Age, Poverty Income Ratio (PIR), Body Mass Index (BMI), Gender, Race, Education, Smoking Exposure, and Alcohol Exposure. BMI was categorized into three groups: normal (BMI <25 kg/m^2^), overweight (25 ≤ BMI ≤ 30 kg/m^2^), and obese (BMI > 30 kg/m^2^), based on participants' BMI values (35). Alcohol consumption was assessed using data from NHANES questionnaires. Participants who had consumed fewer than 12 alcoholic drinks in their lifetime were classified as non-drinkers. Former drinkers were individuals who had consumed ≥12 drinks at any point in their lifetime but had not consumed alcohol in the past year. To minimize recall bias, smoking exposure was evaluated based on serum cotinine levels rather than relying solely on the “smoking history questionnaire”. Current smokers were identified by serum cotinine levels >10 ng/mg, former smokers had serum cotinine levels ≤ 10 ng/mg, and non-smokers exhibited serum cotinine levels <0.011 ng/mg (36).

### 2.5 Statistical analysis

In accordance with Centers for Disease Control and Prevention (CDC) guidelines, our statistical analyses adhered to stipulated principles. To address the complex multi-stage cluster survey design inherent to NHANES, appropriate sample weights were meticulously applied to each participant. Categorical variables were expressed as proportions, while continuous variables were presented as means (mean ± standard deviation). Descriptive statistics comprehensively summarized participants' demographic characteristics and biomarker concentrations. Specifically, for each selected PCB, analysis was conducted after stratifying into three groups based on quartiles, and for substances exhibiting highly right-skewed distributions, analysis was stratified into two groups based on the median.

A total of 69 POPs were initially identified in NHANES data (1999–2004), encompassing dioxins, furans, and PCBs. From these, 18 persistent organic pollutants with valid data representing 75% of the total data were selected for further analysis. WQS regression, focusing on mortality risk, was performed on these 18 substances, with the top seven substances selected based on their cumulative contribution rate to the preceding 95%, all of which were PCBs.

Correlation heatmaps were employed to illustrate the interrelationships among the 7 PCBs and their associations with various diseases. Principal component analysis (PCA) was utilized to visualize the relationship between PCBs and mortality risk in two dimensions. Additionally, five diseases highly correlated with PCBs (Hyperuricemia, Hypertension, Diabetes, CKD, and Arthritis) were included in the machine learning algorithm model, while Cancer, Osteoporosis, and Hepatitis C (HCV) were excluded due to either their broad spectrum or insufficient data volume. Further, multivariable logistic regression was employed to evaluate the associations between these substances and 25 diseases adjusted for Age, Gender, Race, BMI, Social Inequality Index (SII), smoking exposure, and alcohol exposure. We analyzed each of the seven PCBs separately for each disease outcome, calculating the effect of each PCB on a specific disease outcome individually. To illustrate the interrelationships among different diseases, logistic regression and comorbidity network analysis were also performed on the 25 diseases, with the most relevant diseases determined based on Odds Ratios (ORs) and *p*-values, and results presented via network analysis diagrams.

Furthermore, based on the above analysis, we used both traditional Cox regression and machine learning models to investigate whether PCBs act as independent risk factors for mortality. In the Cox regression analysis, after adjusting for baseline and disease data, we found a significant positive correlation between each PCB and mortality risk (HR > 1). Simultaneously, we developed a high-precision mortality risk prediction model. After incorporating PCB data into the baseline and disease data, the model showed a significant improvement in predictive accuracy. The study further demonstrated that PCBs are independent risk factors for mortality, even after adjusting for confounding factors such as diseases. We constructed five individual machine learning models: Support Vector Machine (SVM), Naïve Bayes, Decision Tree, Stochastic Gradient Descent (SGD), and Gradient Boosting Decision Tree (GBDT), as well as four ensemble models: Random Forest, Histogram-based GBDT (Hist GBDT), Bagging, and Neural Networks. Additionally, a voting algorithm was developed for outputting results, which were evaluated using ROC curves and confusion matrices. Moreover, we divided the participants into three groups based on their level of pollutant exposure and then further stratified each group into two subgroups according to whether their DII was >0. Survival curves were plotted for each subgroup, and multivariable Cox regression models were constructed for each group to explore the differential Hazard Ratios (HR) of mortality outcomes associated with the DII, after adjusting for baseline data. Our results effectively demonstrated that dietary patterns modulated by the DII can mitigate the adverse health effects of high PCB exposure in populations with elevated levels of PCB exposure.

## 3 Results

### 3.1 Unveiling the relationship between PCBs and diseases and mortality risk

Table 1, Supplementary Table 1 present the essential characteristics of our study cohort. The mean age of participants was 40.99 years. Regarding gender distribution, females slightly outnumbered males, constituting 50.2% compared to 49.8%. Additionally, we categorized the 10,961 participants into three groups–low, moderate, and high–based on tertiles reflecting PCB concentrations. Notably, individuals in the high LBX074 group displayed distinct characteristics compared to those in the low and moderate groups (Table 1). Specifically, the high LBX074 group exhibited a higher proportion of females, a significantly greater mean age, and a substantially elevated percentage of active smokers. Similar trends were observed for other comprehensive PCB data, as outlined in Supplementary Table 1.

**Table 1:** Baseline demographic characteristics divided by LBX074 levels.

	**Overall**	**LBX074**			***p*-value**
		**Low**	**Moderate**	**High**	
Number	10,961	4,015	3,304	3,642	
Age	40.99 (21.36)	25.80 (12.77)	37.78 (18.11)	61.98 (14.25)	<0.001
**Gender (%)**					<0.001
Male	5,457 (49.8)	2,128 (53.0)	1,847 (55.9)	1,482 (40.7)	
Female	5,504 (50.2)	1,887 (47.0)	1,457 (44.1)	2,160 (59.3)	
**Race (%)**					<0.001
Mexican American	2,692 (24.6)	1,351 (33.6)	824 (24.9)	517 (14.2)	
Non-Hispanic Black	2,353 (21.5)	1,021 (25.4)	679 (20.6)	653 (17.9)	
Non-Hispanic White	5,069 (46.2)	1,295 (32.3)	1,484 (44.9)	2,290 (62.9)	
Other Hispanic	438 (4.0)	176 (4.4)	161 (4.9)	101 (2.8)	
Other races	409 (3.7)	172 (4.3)	156 (4.7)	81 (2.2)	
**Education (%)**					<0.001
<9th grade	1,530 (14.4)	600 (15.4)	439 (13.7)	491 (13.8)	
9–11th grade	1,942 (18.2)	829 (21.3)	533 (16.6)	580 (16.3)	
High-school graduate	2,454 (23.0)	889 (22.9)	748 (23.3)	817 (23.0)	
College graduate or above	1,995 (18.7)	581 (14.9)	673 (20.9)	741 (20.9)	
Some college or AA degree	2,679 (25.1)	968 (24.9)	810 (25.2)	901 (25.4)	
Others	56 (0.5)	20 (0.5)	13 (0.4)	23 (0.6)	
PIR	2.50 (1.60)	2.17 (1.56)	2.61 (1.63)	2.77 (1.56)	<0.001
**BMI stage (%)**					<0.001
<25	4,488 (42.5)	2,158 (55.1)	1,361 (42.1)	969 (28.4)	
>30	2,776 (26.3)	724 (18.5)	852 (26.4)	1,200 (35.1)	
25–30	3,300 (31.2)	1,035 (26.4)	1,020 (31.5)	1,245 (36.5)	
**Smoking exposure (%)**					<0.001
Current smoker	2,596 (23.8)	934 (23.4)	938 (28.6)	724 (20.0)	
Former smoker	6,646 (61.0)	2,529 (63.2)	2,003 (61.1)	2,114 (58.5)	
Non smoker	1,649 (15.1)	537 (13.4)	338 (10.3)	774 (21.4)	
**Alcohol intake (%)**					<0.001
Current drinker	497 (21.0)	136 (23.7)	128 (22.0)	233 (19.3)	
Former drinker	696 (29.4)	107 (18.6)	170 (29.2)	419 (34.7)	
Non drinker	1,173 (49.6)	331 (57.7)	285 (48.9)	557 (46.1)	
SII	582.86 (367.92)	557.14 (340.40)	583.96 (395.89)	610.40 (368.91)	<0.001
CRP	0.38 (0.81)	0.26 (0.56)	0.38 (0.93)	0.52 (0.89)	<0.001
WBC	7.09 (2.18)	7.04 (2.03)	7.18 (2.12)	7.07 (2.39)	0.018
Lymphocyte	2.13 (1.01)	2.18 (0.62)	2.15 (0.67)	2.07 (1.49)	<0.001
Monocyte	0.56 (0.18)	0.55 (0.18)	0.56 (0.18)	0.57 (0.19)	<0.001
Neutrophils	4.16 (1.65)	4.08 (1.68)	4.22 (1.75)	4.18 (1.51)	<0.001
Platelet count	272.08 (67.52)	277.92 (63.12)	274.24 (72.13)	263.63 (67.08)	<0.001
Red blood cell	4.74 (0.50)	4.81 (0.49)	4.79 (0.48)	4.60 (0.51)	<0.001
Hemoglobin	14.28 (1.51)	14.38 (1.53)	14.43 (1.55)	14.05 (1.40)	<0.001
Alkaline phosphatase	90.57 (62.34)	102.04 (76.08)	94.53 (68.44)	74.22 (24.98)	<0.001
Albumin	4.32 (0.33)	4.38 (0.29)	4.39 (0.36)	4.20 (0.30)	<0.001
Bilirubin	0.72 (0.29)	0.74 (0.30)	0.71 (0.33)	0.71 (0.25)	<0.001
Iron	88.34 (37.89)	90.12 (40.00)	90.86 (38.84)	84.06 (34.05)	<0.001

The baseline table is a calculation from NHANES 1999–2000, 2001–2002, 2003–2004. For categorical variables, the p-value was calculated by the chi-square test. For continuous variables, the p-value was calculated by t-test. SII, systemic immune-inflammation index; CRP, C-reactive protein; WBC, white blood cell.

**Figure 1:**
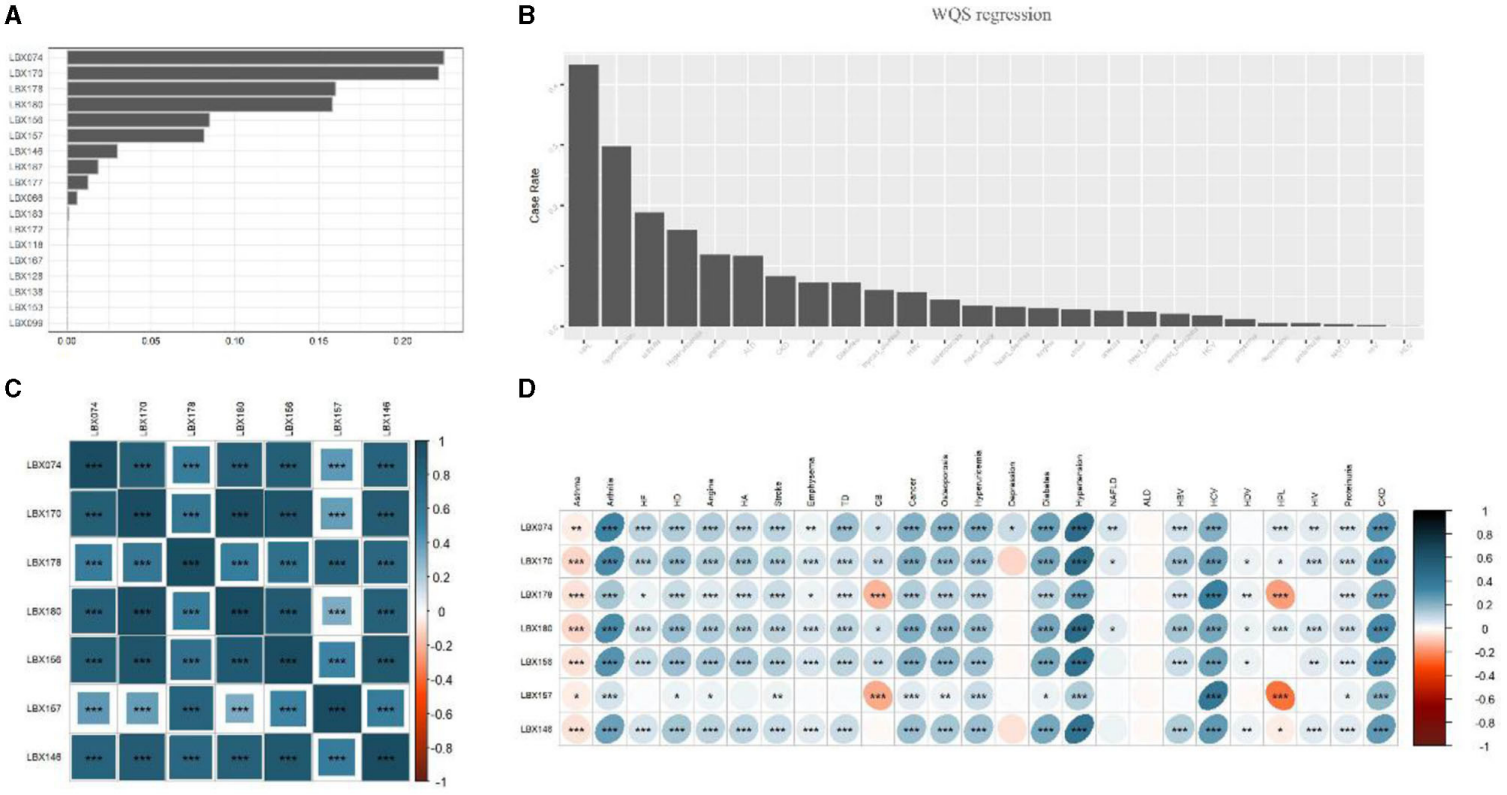
Selection of PCBs and heat map analysis of the relationship between PCBs and disease. **(A)** Progressive weighted quantile and regression (WQS) for POPs with a valid data volume >75%. Seven PCBs with the top 95% cumulative weight were selected. **(B)** The prevalence of each disease was ranked among all participants. **(C)** Correlation heat map analysis of seven types of PCBs. The graph shows that the content of each PCBs in the human body is highly positively correlated. **(D)** Heatmap analysis of the correlation between 7 PCBs and diseases. Lesions were expressed as 1 and 0, both using Spearman correlation analysis. The images showed that the seven PCBs were significantly positively correlated with most diseases (*p* < 0.0001), however, all seven PCBs were negatively correlated with Asthma, and LBX178 and LBX167 were negatively correlated with Chronic bronchitis and Hyperlipidemia. But alcoholic fatty liver is almost unrelated to all PCBs. HA, heart attack; HD, heart disease; HF, heart failure; ALD, alcoholic fatty liver disease; HBV, hepatitis B virus; HCV, hepatitis C virus; HDV, hepatitis D virus; NAFLD, non-alcoholic fatty liver disease; TD, thyroid disease; HIV, human immunodeficiency virus; CB, chronic bronchitis; CKD, chronic kidney disease. ^*^0.001 <*P* ≤ 0.05. ^**^0.0001 <*P* ≤ 0.01. ^***^P ≤ 0.0001.

Figures 1A, B depict the results of WQS and weighting analysis for the 18 PCBs and 25 diseases. By calculating the cumulative contribution rate, we identified the top seven substances, ranked in descending order based on their contribution rate, accounting for 95% of the cumulative contribution rate:

LBX074, representing PCB074(denoting 2,4,4′,5-Tetrachlorobiphenyl).

LBX170, representing PCB170(2,2′,3,3′,4,4′,5-Heptachlorobiphenyl).

LBX178, representing PCB178(2,2′,3,3′,5,5′,6-Heptachlorobiphenyl).

LBX180, representing PCB180(2,2′,3,4,4′,5,5′-Heptachlorobiphenyl).

LBX156, representing PCB156(2,3,3′,4,4′,5-Hexachlorobiphenyl).

LBX157, representing PCB157(2,3,3′,4,4′,5′-Hexachlorobiphenyl).

LBX146, representing PCB146(2,3,3′,4,4′,5′-Hexachlorobiphenyl).

In line with this, the main text primarily focused on LBX074 data, aimed at representing this category of substances, while additional data were predominantly included in the Supplementary material.

Figures 1C, D illustrate, in the form of heatmaps, the interrelationships among the seven PCBs and between PCBs and the 25 diseases, respectively. Significant positive correlations were observed among all seven PCBs (*p* < 0.0001), with LBX146 exhibiting the strongest correlation with the other six substances. The seven PCBs demonstrated significant positive correlations with most diseases, including arthritis, cardiovascular diseases, respiratory disorders, endocrine diseases, chronic hepatitis, and chronic kidney disease (*p* < 0.0001). However, all seven PCBs were negatively correlated with Asthma, and LBX178 and LBX167 were negatively correlated with Chronic bronchitis and Hyperlipidemia. Notably, Alcoholic fatty liver showed almost no correlation with any of the PCBs. These findings suggest that PCBs might be important environmental factors associated with the development of most diseases, affecting multiple systems in the body.

**Figure 2:**
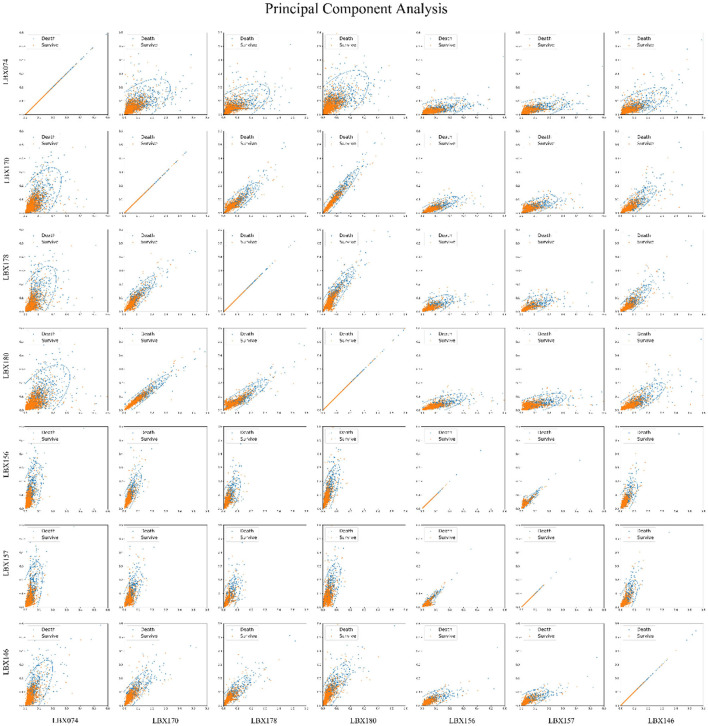
Principal component analysis (PCA) of 7 PCBs and mortality risk. Principal component analysis was used to reduce the dimensionality of the seven-dimensional data that affects the mortality risk, i.e., seven kinds of PCBs, and the seven kinds of PCBs were paired in pairs into two-dimensional graphics. The images show that the higher the PCBs, the greater the mortality risk, and all seven PCBs are important factors that increase the mortality risk.

Figure 2 employed principal component analysis (PCA) to illustrate the relationship between PCBs and the risk of death in a two-dimensional format. Pairwise combinations of the seven PCBs were analyzed, and the PCA two-dimensional plot indicates that for each pair of PCBs, higher PCB levels correspond to increased risk of death. Thus, all seven PCBs are important factors in increasing the mortality risk.

**Figure 3:**
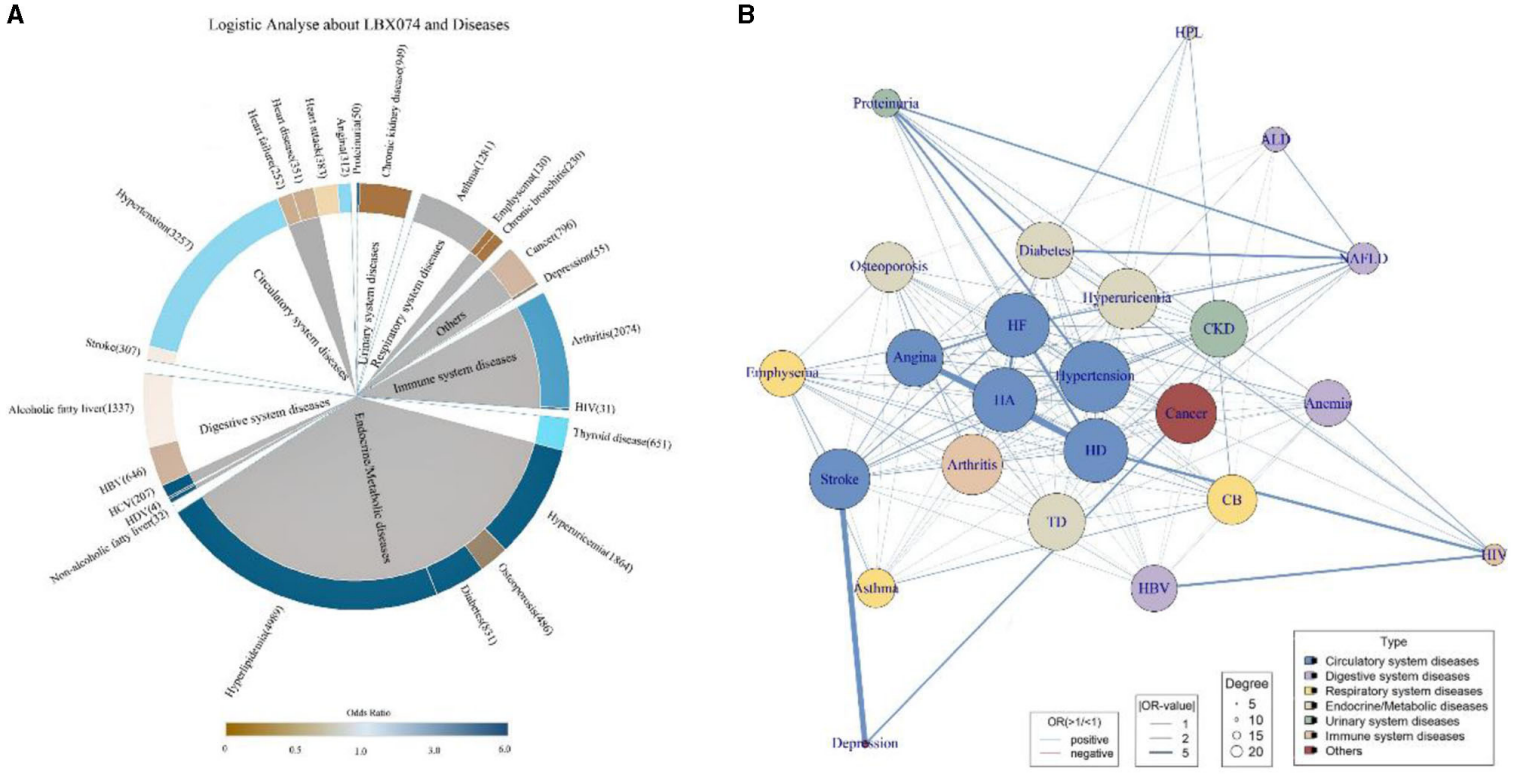
Logistic regression and comorbid network analysis. PCBs are independent influencing factors for a variety of diseases. **(A)** Taking LBX074 as the representative of PCBs, multivariable logistic regression was performed with 25 diseases, and Age, Gender, Race, BMI, SII, smoking exposure and alcohol exposure were adjusted. The color of the inner circle was derived from the *p*-value obtained by logistic regression, and the color of the inner circle was gray when *p* < 0.05, and the color of the inner circle was white when *p* > 0.05. **(B)** 25 disease comorbidity network diagrams. Each node represents a disease, and the width of the link represents the strength of the comorbid association. According to the comorbid network analysis, we constructed 283 possible links, performed logistic regression on the disease pairs, and the OR values and the results of the comorbid network analysis were displayed in the figure. The closer you are to the center of the network, the more central it is, and the more nodes are associated with other diseases. HA, heart attack; HD, heart disease; HF, heart failure; ALD, alcoholic fatty liver disease; HBV, hepatitis B virus; HCV, hepatitis C virus; HDV, hepatitis D virus; NAFLD, non-alcoholic fatty liver disease; TD, thyroid disease; HIV, human immunodeficiency virus; CB, chronic bronchitis; CKD, chronic kidney disease.

Multivariable logistic regression was employed to analyze the summarized 25 diseases, adjusting for Age, Gender, Race, BMI, SII, smoking exposure, and alcohol exposure. Each of the seven PCBs was individually analyzed in relation to each specific disease outcome, with the impact of each PCB on a given disease outcome calculated separately. Among them, 12 diseases showed significant associations with PCBs (*p* < 0.05), with six diseases significantly positively correlated with PCBs (OR>1, *p* < 0.05) (Figure 3A), including Hyperuricemia (OR > 6.0, *p* < 0.001), Diabetes (OR > 6.0, *p* < 0.0001), HCV (OR > 6, *p* = 0.0071), Hyperlipidemia (OR > 6, *p* = 0.016), HIV (OR > 6, *p* = 0.0005), and Arthritis (OR = 5.42, *p* = 0.0065). Adjusting for covariates, it could be demonstrated that PCBs were independent influencing factors for multiple diseases.

Logistic regression was used to compute the odds ratio (OR) and *p*-values for each pair of the 25 diseases to determine disease associations. Each disease outcome was paired with the remaining 24 disease outcomes, and by continuing to adjust for covariates consistent with the previous models, 600 different paired models were generated. We calculated the OR values for all disease pairs (Supplementary Table 2) and presented the disease pairs most closely related to each disease (*p* < 0.05) (Supplementary Table 3). Among these pairs, Depression and Stroke emerged as the most correlated diseases (OR [95%CI]: 40.21[5.83, 794.00], *p* = 0.001), while the association between Thyroid disease and Angina was the least significant (OR [95%CI]: 3.60 [2.76, 4.66], *p* < 0.0001). Diseases of the circulatory system tend to be interrelated, with diseases highly correlated with diabetes and proteinuria, typical of the urinary system, accounting for the majority of associations. Depression is strongly associated with some common chronic clinical conditions. Based on comorbidity network analysis, we identified 283 potential links, with the number of related links, OR values, and comorbidity network analysis results shown in Figure 3B. Each node represents a medical condition, and the thickness of the connecting lines reflects the strength of the disease pairs' association. Nodes closer to the network center have stronger centrality, indicating a greater number of connections with other diseases. Hypertension exhibits the strongest centrality and the highest number of connections, significantly influencing most other diseases. It is also notable that diseases of the circulatory system are often closely associated with other diseases. Depression, ALD, HIV, and HPL are distanced from the center, showing fewer associations with other diseases and smaller impact.

### 3.2 Unveiling the association between PCBs with mortality using machine learning, introducing DII for correction

Before conducting machine learning predictions, we first applied the traditional Cox regression method for analysis (Supplementary Figure 2). The model was adjusted for baseline demographic and disease data, consistent with the data included in the predictive model. This analysis successfully validated the independent effect of PCBs on mortality risk. A significant positive correlation was observed between most PCBs and mortality risk, with increased PCB exposure corresponding to a notable rise in mortality risk. For example, PCB74 (HR [95% CI]: 1.990 [1.288, 3.074], *p* = 0.002, group of “High”), PCB170 (HR [95% CI]: 2.015 [1.039, 3.909], *p* = 0.038, group of “High”), and PCB180 (HR [95% CI]: 3.090 [1.667, 5.729], *p* = 0.0003, group of “High”).

**Figure 4:**
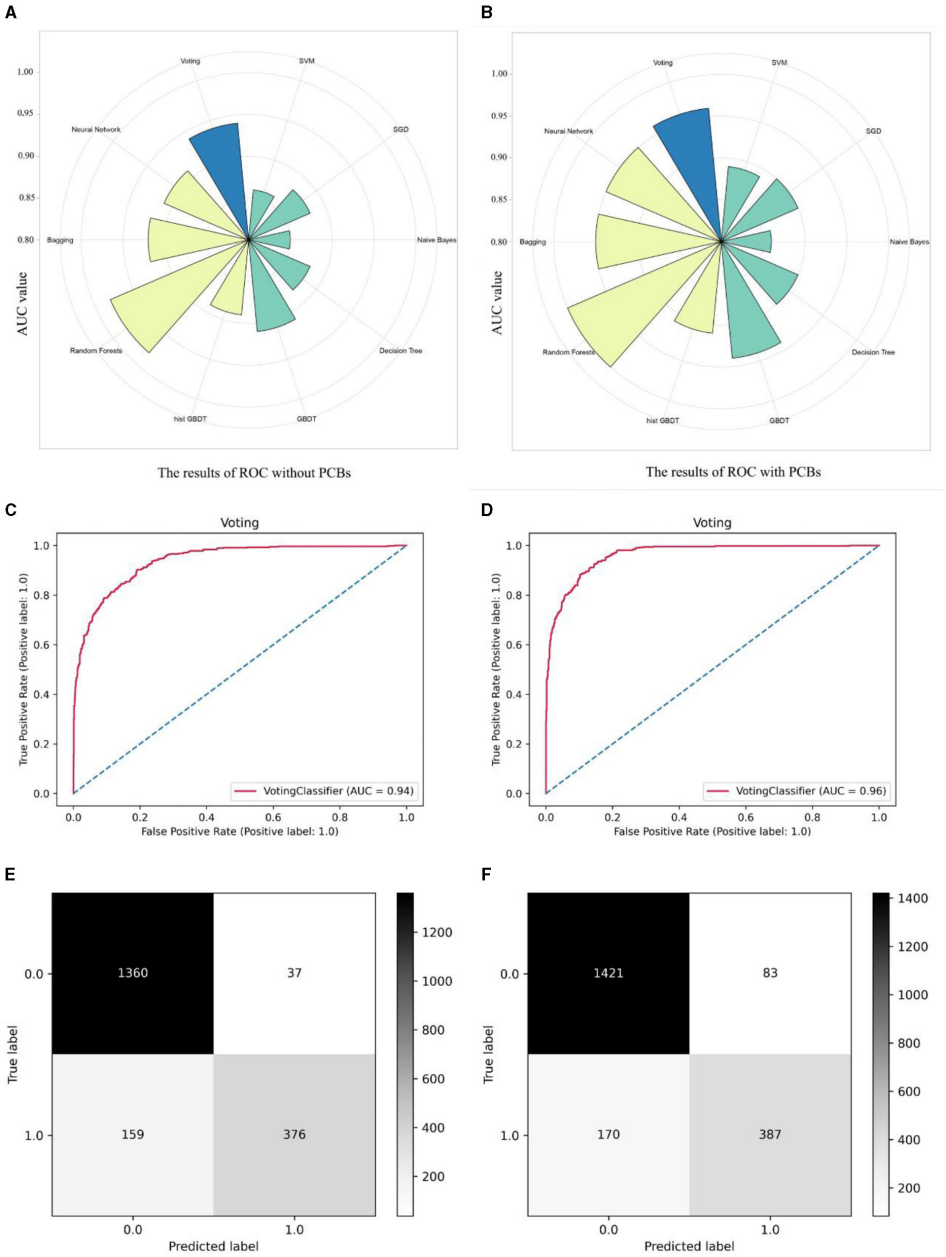
Comparison of the accuracy differences between different machine learning models before and after adding PCBs. Classifying machine learning models into single models and composite models, as well as a Voting model for the final output, radial histograms integrate data from ROC curves across multiple models. **(A)** The data models including population baseline data Age, gender, race and five diseases as hyperuricemia, hypertension, diabetes, CKD, and arthritis. **(B)** The data models including population baseline data Age, gender, race and five diseases as hyperuricemia, hypertension, diabetes, CKD, and arthritis, adding seven PCBs. **(C)** The ROC curve before the PCBs data is included in the Voting algorithm model. **(D)** The ROC curve after the PCBs data is included in the Voting algorithm model. **(E)** The Confusion matrix before the PCBs data is included in the Voting algorithm model. **(F)** The Confusion matrix after the PCBs data is included in the Voting algorithm model.

After that, we constructed nine learning models, including five individual models: Support Vector Machine (SVM), Naïve Bayes, Decision Tree (Tree), Stochastic Gradient Descent (SGD), and Gradient Boosting Decision Tree (GBDT). Additionally, we developed four ensemble models: Random Forest, Histogram Gradient Boosting Decision Tree (hist GBDT), Bagging, and Neural Network. Furthermore, we incorporated a Voting algorithm for result output. Two types of models were added to the algorithm: one containing Age, gender, race, hyperuricemia, hypertension, diabetes, CKD, and arthritis, and the other adding PCBs data. The AUC values of these two types of models, representing predictive accuracy, were compared (Figures 4A, B). It is evident that models incorporating PCBs data exhibited varying degrees of improvement in accuracy compared to the baseline models. Overall, ensemble models outperformed individual models, with Random Forests showing significant advantages in prediction. Prior to incorporating PCBs data, the accuracy rates were as follows: SVM (0.86), SGD (0.88), Naïve Bayes (0.85), Decision Tree (0.88), GBDT (0.91), hist GBDT (0.89), Random Forests (0.98), Bagging (0.92), Neural Network (0.91), and the final Voting model (0.94). After incorporating PCBs data, the accuracy rates were: SVM (0.89), SGD (0.90), Naïve Bayes (0.86), Decision Tree (0.90), GBDT (0.94), hist GBDT (0.91), Random Forests (1.0), Bagging (0.95), Neural Network (0.95), and the final Voting model (0.96). Figures 4C, D illustrate the comparison of ROC curves before and after applying the Voting algorithm. Figures 4E, F illustrate the comparison of the confusion matrices for the Voting algorithm before and after. These results strongly demonstrate a substantive positive correlation between PCBs and mortality risk, even after controlling for baseline data and confounding factors such as diseases. The ROC curves and confusion matrices of the other algorithms are presented in Supplementary Figure 1.

**Figure 5:**
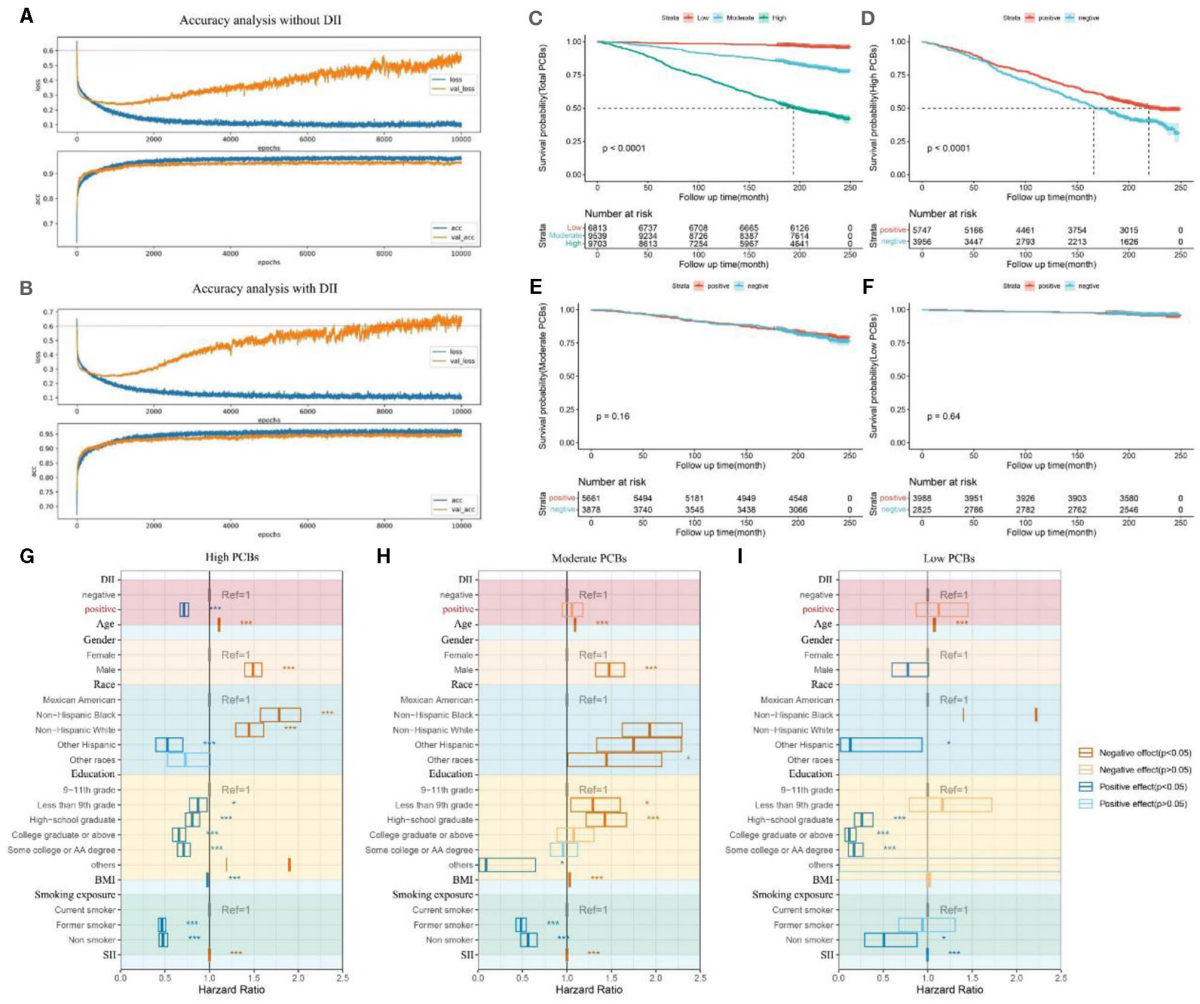
Survival curves of different categories of participants classified according to the total amount of 7 PCBs and the DII index. **(A)** The accuracy of the machine learning prediction model when DII data is not included. The data models including population baseline data Age, gender, race and five diseases as hyperuricemia, hypertension, diabetes, CKD, arthritis, and seven PCBs. **(B)** The accuracy of the machine learning prediction model when DII data is included. The data models including population baseline data Age, gender, race and five diseases as hyperuricemia, hypertension, diabetes, CKD, arthritis, and seven PCBs, adding DII index. **(C)** Survival curves for three types of participants classified according to PCBs. The total amount of 7 PCBs in the participants was calculated and divided into three categories: “Low”, “Moderate” and “High” according to the tripart. **(D)** Survival curves of two types of participants classified according to the DII index. The DII index of the participants was calculated and the DII >0 was defined as “positive” and the DII <0 was defined as “negative”, which was classified among the participants with Total PCBs as “High”. **(E)** Survival curves of two types of participants classified according to the DII index. The DII index of the participants was calculated and the DII >0 was defined as “positive” and the DII <0 was defined as “negative”, which was classified among the participants with Total PCBs as “Moderate”. **(F)** Survival curves of 2 types of participants classified according to the DII index. The DII index of the participants was calculated and the DII >0 was defined as “positive” and the DII <0 was defined as “negative”, which was classified among the participants with Total PCBs as “Low”. **(G)** Multifactorial Cox regression forest plot for participants with high PCBs. DII scores below 1 are denoted as 'positive,' while DII scores above 1 are denoted as 'negative.' Factors adversely affecting health are depicted in red, while factors beneficial to health are depicted in blue. The impact of DII on mortality among the high PCBs population was explored, adjusting for age, gender, race, education, BMI, smoking exposure, and SII. **(H)** Multifactorial Cox regression forest plot for participants with moderate PCBs. **(I)** Multifactorial Cox regression forest plot for participants with high PCBs. DII, dietary inflammatory index; BMI, body mass index; SII, systemic immune-inflammation index. ^*^0.001 <*P* ≤ 0.05. ^**^ 0.0001 <*P* ≤ 0.01. ^***^ *P* ≤ 0.0001.

Furthermore, in Figure 5, we demonstrated through survival curves that we could mitigate the impact of DII by adjusting lifestyle habits. We first continued to utilize the previously built machine learning models to calculate the accuracy and errors of the predictive model containing population baseline data (Age, gender, race) and 5 diseases (hyperuricemia, hypertension, diabetes, CKD, arthritis), along with 7 PCBs data (Figure 5A). Furthermore, we incorporated DII data of each participant into the aforementioned model for prediction and recalculated the accuracy and errors of the new model (Figure 5B). The results indicated that the DII index effectively enhanced the accuracy of the model, highlighting its significance as a contributing factor to increased mortality risk. In Figure 5C, participants were categorized into low, medium, and high groups based on the quartiles of the total amount of seven PCBs in their bodies, and survival curves were plotted accordingly. Participants with high levels of PCBs showed a significantly increased risk of mortality, while participants with medium and low levels exhibited progressively lower risks of mortality (*p* < 0.0001). In Figure 5D, participants with high levels of PCBs were further classified based on their DII scores: those with DII scores >0 were defined as “positive,” while those with DII scores ≤ 0 were defined as “negative.” In comparison with Figure 5C, it is evident that a DII score >0 (indicating reduced inflammation) effectively reduces the risk of mortality among participants with higher levels of PCBs. Figures 5E, F depict similar classifications for participants with medium and low levels of PCBs, respectively. However, the conclusions drawn from participants with high levels of PCBs were not statistically significant in these two groups, indicating no significant differences.

In Figures 5G–I, we further constructed multifactor Cox regression models to examine the association between DII index and mortality, adjusting for age, gender, race, education, BMI, smoking exposure, and SII covariates. Among the high PCBs population, a DII index <1 (indicating inflammation suppression) was significantly negatively correlated with mortality (OR [95% CI] = 0.7151 [0.6683, 0.7651], *p* < 0.05). However, in the medium PCBs and low PCBs populations, the association between a DII index <1 and mortality was not statistically significant (OR [95% CI] = 1.05689 [0.94773, 1.1786], *p* > 0.05) (OR [95% CI] = 1.125 [0.87141, 1.4525], *p* > 0.05).

Through the above analysis, we effectively demonstrated that among the high PCBs population, modulation of dietary habits and adjustment of DII can effectively counteract the adverse effects of PCBs and similar environmental factors on human health. However, in the medium PCBs and low PCBs populations, the impact of DII adjustment on mortality is not evident.

## 4 Discussion

Our study revealed a profound connection between PCBs, disease networks, and mortality risk. By constructing a multidimensional machine learning model and conducting multiple iterations to assess accuracy and error, we propose that PCBs may become specific biomarkers for certain diseases in the future. Additionally, we are the first to suggest that controlling the DII could reduce mortality risk and potentially mitigate the impact of environmental factors.

Taking LBX074 (2,4,4,5-Tetrachlorobiphenyl) as an example, LBX074 exhibited significant positive correlations with seven diseases (OR > 1, *p* < 0.05). PCA revealed a notable increase in mortality risk with increasing levels of LBX074, a trend observed in other PCBs as well. Depression and Stroke emerged as the most relevant disease pair in network analysis (OR [95%CI]: 40.21 [5.83, 794.00], *p* = 0.001). When testing mortality risk using machine learning models, we controlled for confounding factors and comorbidities, and incorporated PCBs data, which significantly improved the prediction accuracy. This further confirmed that PCBs are independent risk factors for mortality. The feasibility of reducing mortality risk by lowering DII was validated through survival curve plotting and multi-factor Cox regression analysis. Although the specific pathogenic mechanisms linking coplanar PCBs to mortality rates remain unclear, our study elucidated associations between PCBs and the development of various diseases, shedding light on the factors contributing to increased mortality rates associated with coplanar PCB exposure.

To the best of our knowledge, our study possesses several notable strengths. It is the first to rectify the limitations of traditional methods using artificial intelligence-based big data models to predict mortality risk associated with PCB exposure. Our findings corroborate those of traditional data analysis, with the addition of PCB data significantly enhancing the predictive accuracy of multiple models compared to those without PCB data, suggesting that various PCBs are independent influencing factors on mortality. Additionally, precise measurements of accuracy and errors through multiple iterations provided further evidence of the detrimental effects of pro-inflammatory diets on the body. Traditional statistical methods have limitations in analyzing mortality risk, as they overlook issues of shared exposure and multifactorial risk confounding, and are unable to effectively address statistical errors. With the further development of artificial intelligence, machine learning-based algorithm models can effectively address these issues, with algorithmic results becoming increasingly accurate over time, capable of handling various data formats in dynamic, large-volume, and complex data environments. Therefore, our study, through further data screening and the construction of predictive models using machine learning algorithms, analyzed the impact of PCBs on mortality risk.

Moreover, this study represents the first attempt to comprehensively investigate the combined effects of PCBs on various diseases and comorbidity networks using comorbidity network analysis. Logistic regression was employed to calculate OR values, while centrality and associated nodes were demonstrated through comorbidity network visualization. Consistent with past research, we found significant positive correlations between PCBs and various diseases such as Hyperuricemia, Diabetes, and Hyperlipidemia. Additionally, the comorbidity network analysis indicated that hypertension is a significant trigger for multiple systemic diseases, with circulatory system diseases often closely associated with various other systemic diseases, exhibiting the strongest centrality. Furthermore, through comorbidity network analysis, we first discovered significant positive correlations between PCB levels and HCV, HIV, and arthritis, likely attributable to PCB-induced inflammation, immune suppression, and apoptosis induction in cartilage cells via ROS-dependent pathways. While the specific mechanisms by which PCBs contribute to various diseases and mortality remain unclear, it is undeniable that PCBs pose significant hazards to human health, serving as independent risk factors for multiple diseases and mortality. The impact weight of PCBs is higher than that of some conventional detection substances, suggesting that PCBs may serve as specific biomarkers for certain diseases, aiding in disease prediction in the future. Moreover, further research is needed on the effects of environmental pollutants on human health.

Furthermore, we have introduced for the first time the concept that adverse effects of pollutants can potentially be counteracted by altering dietary habits. Previous research indicates that higher levels of inflammation lead to an increased risk of mortality. Our study further demonstrates the significant role of DII in triggering inflammation and oxidative stress in the disease and mortality processes. Among populations with high PCB exposure, significant reductions in mortality and morbidity risks can be achieved through DII regulation. However, similar effects were not significant among populations with moderate or low PCB exposure. This suggests that PCBs may induce inflammation to a certain threshold, and DII regulation can effectively suppress their effects. Numerous studies have shown that diet, as the main source of bioactive compounds, can mediate inflammatory responses, with pro-inflammatory diets associated with increased white blood cell counts. Pro-inflammatory diets exhibit significant positive correlations with various diseases, including chronic obstructive pulmonary disease, diabetes, depression, and cardiovascular diseases, while high pro-inflammatory diets can increase the risk of mortality, possibly by increasing white blood cell and CRP levels, thereby inducing various diseases leading to mortality. One possible mechanism is the close relationship between diet and the human gut microbiota. Several animal studies have shown that high-sugar diets lead to obesity, insulin resistance, increased intestinal permeability, and low-grade inflammation. Microbial metabolites (such as SCFA butyrates or tryptophan metabolites) can control various physiological functions in the host, ranging from inflammatory responses to energy metabolism in epithelial cells. Bifidobacteria, Lactobacilli, Clostridia, Bacillus subtilis, and fragile bacilli are closely related to specific immunity via MyD88, transforming growth factor-β, IL-1, IL-6, IL-17, IL-22, γ-PgA, and PSA. Fragile bacilli, plant bifidobacteria, and bifidobacteria can regulate inflammatory responses via TLR, NF-κB, and MyD88. Inflammation is closely related to diseases, and therefore high DII can induce diseases by triggering inflammation, while low DII has the opposite effect. However, specific hypotheses cannot be tested in current studies. Therefore, future longitudinal studies could consider the potential mechanisms by which diet-driven inflammation induces mortality or disease. Similarly, future research could determine whether the use of anti-inflammatory diets (such as increasing leafy vegetables, herbs, spices, and certain fruits) can reduce WBC and CRP levels, decrease morbidity, and reduce mortality risk.

However, this study has certain limitations. Firstly, although we included all persistent organic pollutant data from the NHANES database, the limitations of the database, including insufficient sample size and the narrow focus of the study, meant that we only investigated the seven substances contributing most significantly to mortality, which accounted for 95% of the cumulative contribution. Coincidentally, these seven substances are all PCBs. However, we cannot rule out the potential harms caused by other persistent organic pollutants, such as dioxins, furans, organochlorine pesticides, and other types of PCBs, nor can we exclude the possibility of co-exposure to these substances. This limitation constrains the scope of our study. Secondly, due to the recruitment strategy of this study, we might also miss the research and discussion of some substances whose data were incomplete but important enough. Because the NHANES database was limited and unevenly sampled, the population and regional representation of this study was limited. At the same time, the mechanisms by which they cause multiple diseases leading to increased mortality rates remain unknown. Additionally, DII was not compared with energy-adjusted DII (E-DII), which constructs a reference database for energy-adjusted nutritional scoring based on data from the same 11 countries used to calculate DII. Without access to the unique comparison database, E-DII cannot be calculated, so we were unable to compare it in our study. Furthermore, the ubiquity and complexity of exposure not only necessitate further research on the effects of PCBs but also require further investigation into the prevention and monitoring of PCBs, which may help clinicians better understand and control exposure levels of these organic pollutants. Finally, when using the NHANES database for statistical analysis, we selected multiple variables. Indeed, when lots of variables are tested, associations flourish, most are due to chance, some are merely markers, some are due to common non-investigated factors, and just a few are causal. The non-longitudinal nature of these surveys is not helpful to discern whether the statistical associations are meaningful enough. Therefore, we cannot avoid the analysis bias caused by large databases.

## 5 Conclusion

Our study has demonstrated that PCBs are closely associated with the occurrence and progression of various diseases and act as independent risk factors, highlighting their potential biotoxicity. Modifying dietary patterns, specifically through an anti-inflammatory diet, may help mitigate PCB-induced toxicity. This suggests that PCBs might exert their biotoxic effects by activating inflammatory pathways, offering a potential intervention strategy. However, a more comprehensive analysis is still lacking, and further studies are needed to determine the specific mechanisms involved in this process.

## Data Availability

The original contributions presented in the study are included in the article/Supplementary material, further inquiries can be directed to the corresponding author.
