# Supplementary material for: Exploring the impact of polychlorinated biphenyls on comorbidity and potential mitigation strategies
Source: Front Public Health. 2024 Oct 30;12:1474994. doi: 10.3389/fpubh.2024.1474994 (PMC11557481; doi:10.3389/fpubh.2024.1474994)
Supplement: Supplementary file 1 [file Table_1.docx]

**Supplementary Table 1 Participant characteristics (weighted) from the NHANES 1999-2004.**

**Table S1a. Baseline demographic characteristics divided by LBX170 levels.**

|  | **Overall** | **LBX170** | | | **p-Value** |
| --- | --- | --- | --- | --- | --- |
|  |  | **Low** | **Moderate** | **High** |  |
| Number | 10814 | 3666 | 3550 | 3598 |  |
| Age | 40.96 (21.35) | 22.36 (9.68) | 39.74 (16.84) | 62.48 (13.76) | <0.001 |
| Gender (%) |  |  |  |  | <0.001 |
| Male | 5394 (49.9) | 1643 (44.8) | 1852 (52.2) | 1899 (52.8) |  |
| Female | 5420 (50.1) | 2023 (55.2) | 1698 (47.8) | 1699 (47.2) |  |
| Race (%) |  |  |  |  | <0.001 |
| Mexican American | 2655 (24.6) | 1286 (35.1) | 878 (24.7) | 491 (13.6) |  |
| Non-Hispanic Black | 2320 (21.5) | 928 (25.3) | 703 (19.8) | 689 (19.1) |  |
| Non-Hispanic White | 5000 (46.2) | 1143 (31.2) | 1652 (46.5) | 2205 (61.3) |  |
| Other Hispanic | 431 (4.0) | 174 (4.7) | 150 (4.2) | 107 (3.0) |  |
| Other races | 408 (3.8) | 135 (3.7) | 167 (4.7) | 106 (2.9) |  |
| Education (%) |  |  |  |  | <0.001 |
| Less than 9th grade | 1501 (14.3) | 536 (15.2) | 466 (13.4) | 499 (14.2) |  |
| 9-11th grade | 1911 (18.2) | 721 (20.4) | 565 (16.3) | 625 (17.8) |  |
| High-school graduate | 2421 (23.0) | 837 (23.7) | 778 (22.4) | 806 (23.0) |  |
| College graduate or above | 1974 (18.8) | 554 (15.7) | 743 (21.4) | 677 (19.3) |  |
| Some college or AA degree | 2649 (25.2) | 858 (24.3) | 901 (26.0) | 890 (25.4) |  |
| others | 56 (0.5) | 24 (0.7) | 19 (0.5) | 13 (0.4) |  |
| PIR | 2.50 (1.60) | 2.13 (1.52) | 2.65 (1.64) | 2.74 (1.56) | <0.001 |
| BMI stage (%) |  |  |  |  | <0.001 |
| <25 | 4430 (42.5) | 1993 (55.4) | 1340 (38.9) | 1097 (32.5) |  |
| >30 | 2726 (26.2) | 737 (20.5) | 1023 (29.7) | 966 (28.6) |  |
| 25-30 | 3262 (31.3) | 868 (24.1) | 1078 (31.3) | 1316 (38.9) |  |
| Smoking exposure (%) |  |  |  |  | <0.001 |
| Current smoker | 2568 (23.9) | 702 (19.2) | 987 (28.1) | 879 (24.6) |  |
| Former smoker | 6532 (60.8) | 2430 (66.5) | 2117 (60.2) | 1985 (55.4) |  |
| Non smoker | 1647 (15.3) | 521 (14.3) | 410 (11.7) | 716 (20.0) |  |
| Alcohol intake (%) |  |  |  |  | <0.001 |
| Current drinker | 489 (20.9) | 96 (20.7) | 186 (23.8) | 207 (18.9) |  |
| Former drinker | 692 (29.6) | 82 (17.7) | 205 (26.2) | 405 (37.0) |  |
| Non drinker | 1157 (49.5) | 285 (61.6) | 390 (49.9) | 482 (44.1) |  |
| SII | 583.34 (368.93) | 557.43 (309.80) | 589.74 (365.25) | 603.54 (422.95) | <0.001 |
| CRP | 0.38 (0.81) | 0.27 (0.55) | 0.39 (0.83) | 0.49 (0.98) | <0.001 |
| WBC | 7.10 (2.18) | 7.02 (2.02) | 7.23 (2.08) | 7.04 (2.43) | <0.001 |
| Lymphocyte | 2.13 (1.01) | 2.18 (0.60) | 2.17 (0.71) | 2.05 (1.48) | <0.001 |
| Monocyte | 0.56 (0.19) | 0.55 (0.18) | 0.56 (0.18) | 0.57 (0.20) | <0.001 |
| Neutrophils | 4.16 (1.65) | 4.06 (1.66) | 4.26 (1.67) | 4.16 (1.62) | <0.001 |
| Platelet count | 272.17 (67.67) | 283.13 (65.87) | 274.54 (66.37) | 258.58 (68.48) | <0.001 |
| Red blood cell | 4.74 (0.50) | 4.79 (0.50) | 4.79 (0.48) | 4.63 (0.51) | <0.001 |
| Hemoglobin | 14.29 (1.51) | 14.20 (1.55) | 14.44 (1.52) | 14.22 (1.44) | <0.001 |
| Alkaline phosphatase | 90.45 (62.39) | 106.94 (79.15) | 89.69 (64.12) | 74.28 (26.55) | <0.001 |
| Albumin | 4.32 (0.33) | 4.37 (0.30) | 4.38 (0.35) | 4.22 (0.31) | <0.001 |
| Bilirubin | 0.72 (0.29) | 0.74 (0.31) | 0.71 (0.32) | 0.72 (0.26) | <0.001 |
| Iron | 88.36 (37.92) | 88.22 (40.54) | 89.66 (38.28) | 87.21 (34.59) | 0.024 |

**Table S1b. Baseline demographic characteristics divided by LBX178 levels.**

|  | **Overall** | **LBX178** | | **p-Value** |
| --- | --- | --- | --- | --- |
|  |  | **High** | **Low** |  |
| Number | 10927 | 3215 | 7712 |  |
| Age | 40.96 (21.34) | 51.62 (22.67) | 36.76 (19.24) | <0.001 |
| Gender (%) |  |  |  | <0.001 |
| Male | 5430 (49.7) | 1742 (54.2) | 3688 (47.8) |  |
| Female | 5497(50.3) | 1473(45.8) | 4024(52.2) |  |
| Race (%) |  |  |  | <0.001 |
| Mexican American | 2688 (24.6) | 647 (20.1) | 2041 (26.5) |  |
| Non-Hispanic Black | 2354 (21.5) | 722 (22.5) | 1632 (21.2) |  |
| Non-Hispanic White | 5043 (46.2) | 1613 (50.2) | 3430 (44.5) |  |
| Other Hispanic | 438 (4.0) | 113 (3.5) | 325 (4.2) |  |
| Other races | 404 (3.7) | 120 (3.7) | 284 (3.7) |  |
| Education (%) |  |  |  | <0.001 |
| Less than 9th grade | 1522 (14.3) | 565 (18.0) | 957 (12.8) |  |
| 9-11th grade | 1938 (18.2) | 632 (20.2) | 1306 (17.4) |  |
| High-school graduate | 2450 (23.1) | 740 (23.6) | 1710 (22.8) |  |
| College graduate or above | 1986 (18.7) | 513 (16.4) | 1473 (19.7) |  |
| Some college or AA degree | 2671 (25.1) | 673 (21.5) | 1998 (26.7) |  |
| others | 56 (0.5) | 13 (0.4) | 43 (0.6) |  |
| PIR | 2.50 (1.60) | 2.47 (1.56) | 2.51 (1.61) | 0.195 |
| BMI stage (%) |  |  |  | 0.004 |
| <25 | 4474 (42.5) | 1241 (40.3) | 3233 (43.3) |  |
| >30 | 2766 (26.3) | 807 (26.2) | 1959 (26.3) |  |
| 25-30 | 3294 (31.3) | 1028 (33.4) | 2266 (30.4) |  |
| Smoking exposure (%) |  |  |  | <0.001 |
| Current smoker | 2590 (23.9) | 772 (24.2) | 1818 (23.7) |  |
| Former smoker | 6620 (61.0) | 2026 (63.6) | 4594 (59.9) |  |
| Non-smoker | 1647 (15.2) | 389 (12.2) | 1258 (16.4) |  |
| Alcohol intake (%) |  |  |  | <0.001 |
| Current drinker | 497 (21.0) | 154 (18.1) | 343 (22.7) |  |
| Former drinker | 689 (29.2) | 290 (34.2) | 399 (26.4) |  |
| Non-drinker | 1176 (49.8) | 405 (47.7) | 771 (51.0) |  |
| SII | 581.71 (366.51) | 569.29 (349.77) | 586.85 (373.12) | 0.023 |
| CRP | 0.38 (0.81) | 0.45 (0.99) | 0.35 (0.72) | <0.001 |
| WBC | 7.09 (2.18) | 7.10 (2.46) | 7.09 (2.06) | 0.786 |
| Lymphocyte | 2.13 (1.01) | 2.16 (1.55) | 2.13 (0.66) | 0.145 |
| Monocyte | 0.56 (0.18) | 0.57 (0.20) | 0.55 (0.18) | <0.001 |
| Neutrophils | 4.15 (1.65) | 4.12 (1.55) | 4.17 (1.69) | 0.128 |
| Platelet count | 271.94 (67.52) | 261.98 (65.89) | 276.07 (67.77) | <0.001 |
| Red blood cell | 4.74 (0.50) | 4.70 (0.53) | 4.75 (0.49) | <0.001 |
| Hemoglobin | 14.28 (1.51) | 14.27 (1.45) | 14.29 (1.53) | 0.552 |
| Alkaline phosphatase | 90.57 (62.41) | 92.71 (61.96) | 89.68 (62.57) | 0.021 |
| Albumin | 4.32 (0.33) | 4.35 (0.36) | 4.31 (0.31) | <0.001 |
| Bilirubin | 0.72 (0.29) | 0.66 (0.30) | 0.74 (0.29) | <0.001 |
| Iron | 88.29 (37.89) | 87.01 (34.77) | 88.82 (39.10) | 0.023 |

**Table S1c. Baseline demographic characteristics divided by LBX180 levels.**

|  | **Overall** | **LBX180** | | | **p-Value** |
| --- | --- | --- | --- | --- | --- |
|  |  | **low** | **moderate** | **high** |  |
| Number | 10946 | 3705 | 3592 | 3649 |  |
| Age | 40.99 (21.35) | 21.46 (9.14) | 40.40 (15.79) | 62.79 (13.65) | <0.001 |
| Gender (%) |  |  |  |  | <0.001 |
| Male | 5457 (49.9) | 1622 (43.8) | 1860 (51.8) | 1975 (54.1) |  |
| Female | 5489 (50.1) | 2083 (56.2) | 1732 (48.2) | 1674 (45.9) |  |
| Race (%) |  |  |  |  | <0.001 |
| Mexican American | 2687 (24.5) | 1356 (36.6) | 845 (23.5) | 486 (13.3) |  |
| Non-Hispanic Black | 2354 (21.5) | 917 (24.8) | 736 (20.5) | 701 (19.2) |  |
| Non-Hispanic White | 5060 (46.2) | 1115 (30.1) | 1706 (47.5) | 2239 (61.4) |  |
| Other Hispanic | 436 (4.0) | 178 (4.8) | 149 (4.1) | 109 (3.0) |  |
| Other races | 409 (3.7) | 139 (3.8) | 156 (4.3) | 114 (3.1) |  |
| Education (%) |  |  |  |  | <0.001 |
| Less than 9th grade | 1525 (14.3) | 590 (16.5) | 407 (11.6) | 528 (14.8) |  |
| 9-11th grade | 1939 (18.2) | 754 (21.1) | 553 (15.7) | 632 (17.8) |  |
| High-school graduate | 2446 (23.0) | 875 (24.5) | 754 (21.4) | 817 (23.0) |  |
| College graduate or above | 1995 (18.7) | 489 (13.7) | 820 (23.3) | 686 (19.3) |  |
| Some college or AA degree | 2682 (25.2) | 839 (23.5) | 965 (27.4) | 878 (24.7) |  |
| others | 56 (0.5) | 21 (0.6) | 20 (0.6) | 15 (0.4) |  |
| PIR | 2.50 (1.60) | 2.08 (1.52) | 2.72 (1.64) | 2.72 (1.55) | <0.001 |
| BMI stage (%) |  |  |  |  | <0.001 |
| <25 | 4479 (42.4) | 2024 (55.6) | 1303 (37.6) | 1152 (33.4) |  |
| >30 | 2772 (26.3) | 750 (20.6) | 1039 (30.0) | 983 (28.5) |  |
| 25-30 | 3302 (31.3) | 866 (23.8) | 1127 (32.5) | 1309 (38.0) |  |
| Smoking exposure (%) |  |  |  |  | <0.001 |
| Current smoker | 2595 (23.9) | 696 (18.9) | 990 (27.8) | 909 (25.0) |  |
| Former smoker | 6638 (61.0) | 2537 (68.8) | 2092 (58.8) | 2009 (55.3) |  |
| Non-smoker | 1643 (15.1) | 454 (12.3) | 477 (13.4) | 712 (19.6) |  |
| Alcohol intake (%) |  |  |  |  | <0.001 |
| Current drinker | 495 (21.0) | 105 (22.7) | 189 (22.7) | 201 (18.9) |  |
| Former drinker | 691 (29.3) | 70 (15.2) | 228 (27.3) | 393 (36.9) |  |
| Non-drinker | 1176 (49.8) | 287 (62.1) | 417 (50.0) | 472 (44.3) |  |
| SII | 582.94 (368.20) | 558.56 (310.22) | 585.70 (353.32) | 605.12 (430.52) | <0.001 |
| CRP | 0.38 (0.81) | 0.27 (0.57) | 0.38 (0.70) | 0.48 (1.06) | <0.001 |
| WBC | 7.09 (2.18) | 7.06 (2.02) | 7.20 (2.07) | 7.02 (2.43) | 0.001 |
| Lymphocyte | 2.13 (1.01) | 2.20 (0.61) | 2.16 (0.70) | 2.04 (1.47) | <0.001 |
| Monocyte | 0.56 (0.19) | 0.55 (0.18) | 0.56 (0.18) | 0.57 (0.20) | <0.001 |
| Neutrophils | 4.16 (1.65) | 4.08 (1.67) | 4.24 (1.67) | 4.15 (1.61) | <0.001 |
| Platelet count | 272.11 (67.61) | 284.54 (65.85) | 273.21 (66.06) | 258.33 (68.34) | <0.001 |
| Red blood cell | 4.74 (0.50) | 4.77 (0.49) | 4.80 (0.48) | 4.63 (0.52) | <0.001 |
| Hemoglobin | 14.28 (1.51) | 14.14 (1.52) | 14.49 (1.53) | 14.23 (1.44) | <0.001 |
| Alkaline phosphatase | 90.50 (62.32) | 112.71 (84.42) | 83.64 (53.46) | 74.62 (28.05) | <0.001 |
| Albumin | 4.32 (0.33) | 4.39 (0.31) | 4.35 (0.34) | 4.23 (0.31) | <0.001 |
| Bilirubin | 0.72 (0.29) | 0.71 (0.31) | 0.72 (0.30) | 0.73 (0.27) | 0.03 |
| Iron | 88.31 (37.90) | 87.72 (40.65) | 89.44 (37.11) | 87.80 (35.67) | 0.092 |

**Table S1d. Baseline demographic characteristics divided by LBX156 levels.**

|  | **Overall** | **LBX156** | | | **p-Value** |
| --- | --- | --- | --- | --- | --- |
|  |  | **Low** | **Moderate** | **High** |  |
| Number | 10892 | 5084 | 2252 | 3556 |  |
| Age | 41.01 (21.35) | 27.18 (13.55) | 40.73 (19.22) | 62.38 (13.49) | <0.001 |
| Gender (%) |  |  |  |  | <0.001 |
| Male | 5425 (49.8) | 2431 (47.8) | 1195 (53.1) | 1799 (50.6) |  |
| Female | 5440 (50.2) | 2653 (52.2) | 1057 (46.9) | 1757 (49.4) |  |
| Race (%) |  |  |  |  | <0.001 |
| Mexican American | 2681 (24.6) | 1635 (32.2) | 635 (28.2) | 411 (11.6) |  |
| Non-Hispanic Black | 2335 (21.4) | 1221 (24.0) | 445 (19.8) | 669 (18.8) |  |
| Non-Hispanic White | 5037 (46.2) | 1761 (34.6) | 979 (43.5) | 2297 (64.6) |  |
| Other Hispanic | 432 (4.0) | 243 (4.8) | 84 (3.7) | 105 (3.0) |  |
| Other races | 407 (3.7) | 224 (4.4) | 109 (4.8) | 74 (2.1) |  |
| Education (%) |  |  |  |  | 0.001 |
| Less than 9th grade | 1516 (14.3) | 732 (14.8) | 312 (14.3) | 472 (13.6) |  |
| 9-11th grade | 1930 (18.2) | 935 (18.9) | 391 (17.9) | 604 (17.4) |  |
| High-school graduate | 2429 (22.9) | 1136 (23.0) | 452 (20.7) | 841 (24.2) |  |
| College graduate or above | 1993 (18.8) | 851 (17.2) | 439 (20.1) | 703 (20.3) |  |
| Some college or AA degree | 2666 (25.2) | 1249 (25.3) | 583 (26.7) | 834 (24.0) |  |
| others | 56 (0.5) | 32 (0.6) | 9 (0.4) | 15 (0.4) |  |
| PIR | 2.50 (1.60) | 2.29 (1.58) | 2.57 (1.64) | 2.77 (1.56) | <0.001 |
| BMI stage (%) |  |  |  |  | <0.001 |
| <25 | 4462 (42.5) | 2547 (51.3) | 831 (37.8) | 1084 (32.5) |  |
| >30 | 2752 (26.2) | 1101 (22.2) | 664 (30.2) | 987 (29.6) |  |
| 25-30 | 3285 (31.3) | 1314 (26.5) | 706 (32.1) | 1265 (37.9) |  |
| Smoking exposure (%) |  |  |  |  | <0.001 |
| Current smoker | 2575 (23.8) | 1109 (21.9) | 599 (26.9) | 867 (24.6) |  |
| Former smoker | 6606 (61.0) | 3195 (63.1) | 1419 (63.7) | 1992 (56.5) |  |
| Non-smoker | 1641 (15.2) | 763 (15.1) | 211 (9.5) | 667 (18.9) |  |
| Alcohol intake (%) |  |  |  |  | <0.001 |
| Current drinker | 497 (21.1) | 177 (21.6) | 119 (25.6) | 201 (18.7) |  |
| Former drinker | 689 (29.2) | 172 (21.0) | 134 (28.9) | 383 (35.6) |  |
| Non-drinker | 1173 (49.7) | 469 (57.3) | 211 (45.5) | 493 (45.8) |  |
| SII | 583.19 (368.47) | 567.12 (337.04) | 580.10 (338.20) | 608.28 (424.69) | <0.001 |
| CRP | 0.38 (0.81) | 0.30 (0.64) | 0.38 (0.62) | 0.50 (1.07) | <0.001 |
| WBC | 7.09 (2.18) | 7.04 (2.02) | 7.23 (2.12) | 7.07 (2.42) | 0.001 |
| Lymphocyte | 2.13 (1.01) | 2.16 (0.62) | 2.18 (0.72) | 2.06 (1.49) | <0.001 |
| Monocyte | 0.56 (0.19) | 0.55 (0.18) | 0.56 (0.18) | 0.57 (0.20) | <0.001 |
| Neutrophils | 4.15 (1.65) | 4.09 (1.66) | 4.24 (1.72) | 4.19 (1.58) | <0.001 |
| Platelet count | 272.12 (67.58) | 280.05 (65.30) | 274.82 (69.63) | 259.00 (67.53) | <0.001 |
| Red blood cell | 4.74 (0.50) | 4.79 (0.49) | 4.79 (0.48) | 4.62 (0.51) | <0.001 |
| Hemoglobin | 14.28 (1.51) | 14.27 (1.55) | 14.45 (1.52) | 14.19 (1.41) | <0.001 |
| Alkaline phosphatase | 90.54 (62.41) | 98.77 (72.87) | 97.49 (71.18) | 74.27 (26.87) | <0.001 |
| Albumin | 4.32 (0.33) | 4.36 (0.30) | 4.43 (0.35) | 4.21 (0.31) | <0.001 |
| Bilirubin | 0.72 (0.29) | 0.74 (0.30) | 0.67 (0.33) | 0.72 (0.26) | <0.001 |
| Iron | 88.34 (37.89) | 88.88 (40.16) | 89.03 (37.50) | 87.11 (34.60) | 0.065 |

**Table S1e. Baseline demographic characteristics divided by LBX157 levels.**

|  | **Overall** | **LBX157** | | **p-Value** |
| --- | --- | --- | --- | --- |
|  |  | **High** | **Low** |  |
| Number | 10855 | 9023 | 1832 |  |
| Age | 41.03 (21.35) | 41.81 (21.32) | 37.24 (21.11) | <0.001 |
| Gender (%) |  |  |  | 0.884 |
| Male | 5400 (49.7) | 4492 (49.8) | 908 (49.6) |  |
| Female | 5455 (50.3) | 4531 (50.2) | 924 (50.4) |  |
| Race (%) |  |  |  | <0.001 |
| Mexican American | 2671 (24.6) | 2199 (24.4) | 472 (25.8) |  |
| Non-Hispanic Black | 2320 (21.4) | 1892 (21.0) | 428 (23.4) |  |
| Non-Hispanic White | 5026 (46.3) | 4253 (47.1) | 773 (42.2) |  |
| Other Hispanic | 432 (4.0) | 364 (4.0) | 68 (3.7) |  |
| Other races | 406 (3.7) | 315 (3.5) | 91 (5.0) |  |
| Education (%) |  |  |  | 0.004 |
| Less than 9th grade | 1513 (14.3) | 1285 (14.6) | 228 (12.9) |  |
| 9-11th grade | 1920 (18.2) | 1627 (18.5) | 293 (16.6) |  |
| High-school graduate | 2421 (22.9) | 1990 (22.6) | 431 (24.4) |  |
| College graduate or above | 1986 (18.8) | 1668 (19.0) | 318 (18.0) |  |
| Some college or AA degree | 2657 (25.2) | 2179 (24.8) | 478 (27.1) |  |
| others | 56 (0.5) | 40 (0.5) | 16 (0.9) |  |
| PIR | 2.50 (1.60) | 2.53 (1.59) | 2.35 (1.61) | <0.001 |
| BMI stage (%) |  |  |  | 0.076 |
| <25 | 4443 (42.5) | 3644 (42.1) | 799 (44.4) |  |
| >30 | 2749 (26.3) | 2271 (26.2) | 478 (26.5) |  |
| 25-30 | 3270 (31.3) | 2746 (31.7) | 524 (29.1) |  |
| Smoking exposure (%) |  |  |  | 0.021 |
| Current smoker | 2565 (23.8) | 2173 (24.3) | 392 (21.5) |  |
| Former smoker | 6585 (61.1) | 5421 (60.5) | 1164 (63.7) |  |
| Non-smoker | 1635 (15.2) | 1364 (15.2) | 271 (14.8) |  |
| Alcohol intake (%) |  |  |  | 0.329 |
| Current drinker | 494 (21.0) | 411 (20.5) | 83 (23.4) |  |
| Former drinker | 688 (29.2) | 594 (29.7) | 94 (26.6) |  |
| Non-drinker | 1174 (49.8) | 997 (49.8) | 177 (50.0) |  |
| SII | 582.95 (366.92) | 584.24 (353.81) | 576.62 (425.73) | 0.419 |
| CRP | 0.38 (0.80) | 0.39 (0.78) | 0.35 (0.89) | 0.047 |
| WBC | 7.09 (2.18) | 7.09 (2.10) | 7.10 (2.52) | 0.813 |
| Lymphocyte | 2.13 (1.01) | 2.13 (0.89) | 2.15 (1.44) | 0.439 |
| Monocyte | 0.56 (0.18) | 0.56 (0.18) | 0.53 (0.20) | <0.001 |
| Neutrophils | 4.15 (1.65) | 4.15 (1.62) | 4.17 (1.75) | 0.657 |
| Platelet count | 272.10 (67.55) | 272.69 (67.22) | 269.19 (69.10) | 0.044 |
| Red blood cell | 4.74 (0.50) | 4.74 (0.50) | 4.72 (0.49) | 0.18 |
| Hemoglobin | 14.29 (1.50) | 14.28 (1.52) | 14.30 (1.44) | 0.674 |
| Alkaline phosphatase | 90.38 (62.16) | 89.08 (59.04) | 96.77 (75.37) | <0.001 |
| Albumin | 4.32 (0.33) | 4.32 (0.32) | 4.31 (0.34) | 0.238 |
| Bilirubin | 0.72 (0.29) | 0.71 (0.29) | 0.75 (0.33) | <0.001 |
| Iron | 88.34 (37.91) | 89.00 (38.41) | 85.10 (35.20) | <0.001 |

**Table S1f. Baseline demographic characteristics divided by LBX146 levels.**

|  | **Overall** | **LBX146** | | | **p-Value** |
| --- | --- | --- | --- | --- | --- |
|  |  | **Low** | **Moderate** | **High** |  |
| Number | 10925 | 5678 | 1617 | 3630 |  |
| Age | 40.95 (21.35) | 29.66 (15.25) | 37.95 (20.29) | 61.23 (14.86) | <0.001 |
| Gender (%) |  |  |  |  | 0.001 |
| Male | 5431 (49.7) | 2743 (48.3) | 863 (53.4) | 1825 (50.3) |  |
| Female | 5494 (50.3) | 2935 (51.7) | 754 (46.6) | 1805 (49.7) |  |
| Race (%) |  |  |  |  | <0.001 |
| Mexican American | 2687 (24.6) | 1722 (30.3) | 478 (29.6) | 487 (13.4) |  |
| Non-Hispanic Black | 2353 (21.5) | 1257 (22.1) | 279 (17.3) | 817 (22.5) |  |
| Non-Hispanic White | 5046 (46.2) | 2257 (39.7) | 713 (44.1) | 2076 (57.2) |  |
| Other Hispanic | 435 (4.0) | 257 (4.5) | 64 (4.0) | 114 (3.1) |  |
| Other races | 404 (3.7) | 185 (3.3) | 83 (5.1) | 136 (3.7) |  |
| Education (%) |  |  |  |  | 0.003 |
| Less than 9th grade | 1523 (14.3) | 735 (13.3) | 265 (16.9) | 523 (14.8) |  |
| 9-11th grade | 1935 (18.2) | 1008 (18.3) | 282 (18.0) | 645 (18.2) |  |
| High-school graduate | 2448 (23.1) | 1267 (23.0) | 360 (22.9) | 821 (23.2) |  |
| College graduate or above | 1987 (18.7) | 1048 (19.0) | 251 (16.0) | 688 (19.4) |  |
| Some college or AA degree | 2671 (25.2) | 1420 (25.8) | 410 (26.1) | 841 (23.8) |  |
| others | 56 (0.5) | 32 (0.6) | 3 (0.2) | 21 (0.6) |  |
| PIR | 2.50 (1.60) | 2.39 (1.60) | 2.50 (1.64) | 2.67 (1.55) | <0.001 |
| BMI stage (%) |  |  |  |  | <0.001 |
| <25 | 4475 (42.5) | 2754 (49.8) | 648 (40.8) | 1073 (31.4) |  |
| >30 | 2764 (26.2) | 1239 (22.4) | 456 (28.7) | 1069 (31.3) |  |
| 25-30 | 3293 (31.3) | 1537 (27.8) | 486 (30.6) | 1270 (37.2) |  |
| Smoking exposure (%) |  |  |  |  | <0.001 |
| Current smoker | 2590 (23.9) | 1336 (23.6) | 396 (24.9) | 858 (23.8) |  |
| Former smoker | 6617 (61.0) | 3452 (61.1) | 1099 (69.0) | 2066 (57.2) |  |
| Non-smoker | 1648 (15.2) | 865 (15.3) | 98 (6.2) | 685 (19.0) |  |
| Alcohol intake (%) |  |  |  |  | <0.001 |
| Current drinker | 497 (21.1) | 211 (22.7) | 76 (26.2) | 210 (18.4) |  |
| Former drinker | 689 (29.2) | 212 (22.8) | 64 (22.1) | 413 (36.3) |  |
| Non-drinker | 1174 (49.7) | 508 (54.6) | 150 (51.7) | 516 (45.3) |  |
| SII | 581.65 (366.52) | 574.29 (338.62) | 578.72 (431.80) | 594.55 (376.57) | 0.032 |
| CRP | 0.38 (0.81) | 0.30 (0.58) | 0.44 (1.16) | 0.48 (0.90) | <0.001 |
| WBC | 7.09 (2.18) | 7.08 (2.04) | 7.29 (2.17) | 7.02 (2.40) | <0.001 |
| Lymphocyte | 2.13 (1.01) | 2.16 (0.63) | 2.20 (0.71) | 2.07 (1.49) | <0.001 |
| Monocyte | 0.56 (0.18) | 0.55 (0.18) | 0.57 (0.18) | 0.56 (0.20) | 0.005 |
| Neutrophils | 4.15 (1.65) | 4.13 (1.67) | 4.28 (1.79) | 4.14 (1.54) | 0.005 |
| Platelet count | 271.94 (67.51) | 279.95 (65.17) | 271.82 (69.45) | 259.37 (68.34) | <0.001 |
| Red blood cell | 4.74 (0.50) | 4.78 (0.49) | 4.79 (0.47) | 4.64 (0.52) | <0.001 |
| Hemoglobin | 14.28 (1.51) | 14.31 (1.55) | 14.44 (1.43) | 14.18 (1.46) | <0.001 |
| Alkaline phosphatase | 90.57 (62.41) | 95.69 (70.20) | 106.40 (78.98) | 75.43 (28.96) | <0.001 |
| Albumin | 4.32 (0.33) | 4.35 (0.30) | 4.48 (0.37) | 4.21 (0.31) | <0.001 |
| Bilirubin | 0.72 (0.29) | 0.74 (0.30) | 0.65 (0.34) | 0.72 (0.25) | <0.001 |
| Iron | 88.28 (37.88) | 89.37 (40.47) | 88.60 (36.02) | 86.42 (34.26) | 0.001 |
