# Supplementary material for: Exploring the impact of polychlorinated biphenyls on comorbidity and potential mitigation strategies
Source: Front Public Health. 2024 Oct 30;12:1474994. doi: 10.3389/fpubh.2024.1474994 (PMC11557481; doi:10.3389/fpubh.2024.1474994)
Supplement: Supplementary file 2 [file Table_2.docx]

**Supplementary Table 2 All disease pairs' OR values and effectiveness status.**

| **0R** | Asthma | Anemia | Arthritis | HF | HD | Angina | HA | Stroke | Emphysema | TD | CB | Cancer | Osteoporosis | Hyperuricemia | Depression | Diabetes | Hypertension | NAFLD | ALD | HBV | HCV | HPL | HIV | Proteinuria | CKD |
| --- | --- | --- | --- | --- | --- | --- | --- | --- | --- | --- | --- | --- | --- | --- | --- | --- | --- | --- | --- | --- | --- | --- | --- | --- | --- |
| **Asthma** | NA | 1.2987 | 1.4565 | 1.4011 | 1.5207 | 1.7942 | 1.9770 | 1.5210 | 4.8186 | 1.2970 | 5.8510 | 1.0085 | 1.3413 | 1.1947 | 0.6212 | 1.0686 | 1.0561 | 1.5198 | 1.0762 | 0.9050 | 1.3742 | 0.9313 | 0.0000 | 0.5496 | 0.9298 |
| **Anemia** | 1.2987 | NA | 1.9786 | 5.5029 | 1.3656 | 1.8801 | 1.8569 | 2.1035 | 1.8421 | 2.2209 | 2.2119 | 2.1825 | 1.4722 | 1.0526 | 0.0000 | 1.2885 | 1.4281 | 1.9369 | 0.7894 | 1.6365 | 1.9850 | 1.9070 | 6.5314 | 4.4860 | 2.3341 |
| **Arthritis** | 1.4565 | 1.9786 | NA | 3.3860 | 2.1192 | 2.9889 | 2.9887 | 2.8847 | 4.2914 | 2.6974 | 2.4105 | 2.6478 | 6.2732 | 1.7889 | 2.0588 | 2.1645 | 3.3641 | 0.9104 | 0.8179 | 1.0791 | 0.7260 | 1.4490 | 0.0000 | 0.4734 | 2.7858 |
| **HF** | 1.4011 | 5.5029 | 3.3860 | NA | 18.0072 | 13.7974 | 22.2624 | 8.8315 | 5.3680 | 1.0973 | 2.5837 | 3.1495 | 2.5521 | 3.4101 | 0.0000 | 3.3256 | 6.3731 | 9.7096 | 0.9653 | 1.4870 | 0.7207 | 1.4887 | 0.0000 | 4.3394 | 4.1660 |
| **HD** | 1.5207 | 1.3656 | 2.1192 | 18.0072 | NA | 35.2447 | 37.9189 | 5.0485 | 3.2415 | 2.0664 | 1.4753 | 2.3776 | 1.9154 | 2.8576 | 0.0000 | 3.6692 | 4.7422 | 4.6551 | 0.8646 | 1.4680 | 0.5445 | 1.3067 | 20.2903 | 3.5895 | 3.4954 |
| **Angina** | 1.7942 | 1.8801 | 2.9889 | 13.7974 | 35.2447 | NA | 31.9529 | 5.4084 | 2.7876 | 3.6033 | 2.5886 | 2.3043 | 3.5368 | 2.6860 | NA | 3.3192 | 5.3175 | 2.4212 | 0.5858 | 1.1860 | 0.4100 | 1.2832 | 0.0000 | 3.4874 | 3.9484 |
| **HA** | 1.9770 | 1.8569 | 2.9887 | 22.2624 | 37.9189 | 31.9529 | NA | 7.9246 | 2.8888 | 1.9527 | 3.0466 | 2.1267 | 1.8668 | 2.3727 | 0.0000 | 2.9166 | 4.1831 | 3.3220 | 0.8258 | 1.4390 | 0.6483 | 2.0434 | 0.0000 | 2.4840 | 4.3085 |
| **Stroke** | 1.5210 | 2.1035 | 2.8847 | 8.8315 | 5.0485 | 5.4084 | 7.9246 | NA | 6.3355 | 1.7523 | 1.3545 | 2.2664 | 1.7406 | 1.6550 | 40.2143 | 3.3430 | 4.1699 | 2.3446 | 0.8956 | 2.2887 | 1.1335 | 0.9845 | 0.0000 | 1.9685 | 2.6438 |
| **Emphysema** | 4.8186 | 1.8421 | 4.2914 | 5.3680 | 3.2415 | 2.7876 | 2.8888 | 6.3355 | NA | 1.6774 | 4.3449 | 2.9737 | 3.7504 | 1.1572 | NA | 1.2876 | 3.3930 | 0.0000 | 1.2789 | 3.1222 | 0.9555 | 0.4794 | 0.0000 | 0.0000 | 1.7616 |
| **TD** | 1.2970 | 2.2209 | 2.6974 | 1.0973 | 2.0664 | 3.6033 | 1.9527 | 1.7523 | 1.6774 | NA | 2.7552 | 1.6201 | 3.8492 | 1.2930 | 0.5454 | 1.8714 | 2.1638 | 0.9380 | 0.8274 | 0.6024 | 0.6372 | 1.6955 | 0.0000 | 0.0000 | 2.1429 |
| **CB** | 5.8510 | 2.2119 | 2.4105 | 2.5837 | 1.4753 | 2.5886 | 3.0466 | 1.3545 | 4.3449 | 2.7552 | NA | 0.6911 | 2.9434 | 1.3232 | 0.6894 | 1.0984 | 1.5691 | 0.0000 | 0.6005 | 0.5110 | 0.3206 | 5.1809 | 0.0000 | 0.3981 | 0.5544 |
| **Cancer** | 1.0085 | 2.1825 | 2.6478 | 3.1495 | 2.3776 | 2.3043 | 2.1267 | 2.2664 | 2.9737 | 1.6201 | 0.6911 | NA | 2.8982 | 1.5142 | 13.3571 | 1.9465 | 3.1919 | 3.3386 | 0.8573 | 0.6720 | 1.6015 | 1.1045 | 0.0000 | 1.4625 | 3.4401 |
| **Osteoporosis** | 1.3413 | 1.4722 | 6.2732 | 2.5521 | 1.9154 | 3.5368 | 1.8668 | 1.7406 | 3.7504 | 3.8492 | 2.9434 | 2.8982 | NA | 1.0077 | 0.0000 | 1.1756 | 3.4190 | 0.8600 | 0.6198 | 0.9578 | 0.1707 | 2.6470 | 0.0000 | 0.0000 | 3.0295 |
| **Hyperuricemia** | 1.1947 | 1.0526 | 1.7889 | 3.4101 | 2.8576 | 2.6860 | 2.3727 | 1.6550 | 1.1572 | 1.2930 | 1.3232 | 1.5142 | 1.0077 | NA | 0.3742 | 1.6031 | 2.8882 | 2.3249 | 2.1333 | 1.1748 | 1.6662 | 2.0593 | 2.3660 | 4.6384 | 3.8434 |
| **Depression** | 0.6212 | 0.0000 | 2.0588 | 0.0000 | 0.0000 | NA | 0.0000 | 40.2143 | NA | 0.5454 | 0.6894 | 13.3571 | 0.0000 | 0.3742 | NA | 0.0000 | 0.9883 | 0.0000 | 0.3332 | 0.0000 | 0.0000 | NA | 0.0000 | NA | 0.0000 |
| **Diabetes** | 1.0686 | 1.2885 | 2.1645 | 3.3256 | 3.6692 | 3.3192 | 2.9166 | 3.3430 | 1.2876 | 1.8714 | 1.0984 | 1.9465 | 1.1756 | 1.6031 | 0.0000 | NA | 5.1942 | 17.3750 | 1.0990 | 1.5681 | NA | 5.2121 | 6.6534 | 17.9795 | 2.7337 |
| **Hypertension** | 1.0561 | 1.4281 | 3.3641 | 6.3731 | 4.7422 | 5.3175 | 4.1831 | 4.1699 | 3.3930 | 2.1638 | 1.5691 | 3.1919 | 3.4190 | 2.8882 | 0.9883 | 5.1942 | NA | 5.0288 | 1.0369 | 1.3675 | 1.3618 | 2.2162 | 2.5371 | 16.9289 | 5.2341 |
| **NAFLD** | 1.5198 | 1.9369 | 0.9104 | 9.7096 | 4.6551 | 2.4212 | 3.3220 | 2.3446 | 0.0000 | 0.9380 | 0.0000 | 3.3386 | 0.8600 | 2.3249 | 0.0000 | 17.3750 | 5.0288 | NA | 6.7863 | 0.9550 | 1.9324 | 5.9661 | 0.0000 | 14.0714 | 3.4147 |
| **ALD** | 1.0762 | 0.7894 | 0.8179 | 0.9653 | 0.8646 | 0.5858 | 0.8258 | 0.8956 | 1.2789 | 0.8274 | 0.6005 | 0.8573 | 0.6198 | 2.1333 | 0.3332 | 1.0990 | 1.0369 | 6.7863 | NA | 1.4154 | 7.3478 | 1.0269 | 1.7926 | 0.8444 | 1.1444 |
| **HBV** | 0.9050 | 1.6365 | 1.0791 | 1.4870 | 1.4680 | 1.1860 | 1.4390 | 2.2887 | 3.1222 | 0.6024 | 0.5110 | 0.6720 | 0.9578 | 1.1748 | 0.0000 | 1.5681 | 1.3675 | 0.9550 | 1.4154 | NA | 9.8326 | 1.6822 | 17.6482 | 1.2557 | 1.1995 |
| **HCV** | 1.3742 | 1.9850 | 0.7260 | 0.7207 | 0.5445 | 0.4100 | 0.6483 | 1.1335 | 0.9555 | 0.6372 | 0.3206 | 1.6015 | 0.1707 | 1.6662 | 0.0000 | NA | 1.3618 | 1.9324 | 7.3478 | 9.8326 | NA | 0.0000 | 14.1231 | 9.5311 | 0.4129 |
| **HPL** | 0.9313 | 1.9070 | 1.4490 | 1.4887 | 1.3067 | 1.2832 | 2.0434 | 0.9845 | 0.4794 | 1.6955 | 5.1809 | 1.1045 | 2.6470 | 2.0593 | NA | 5.2121 | 2.2162 | 5.9661 | 1.0269 | 1.6822 | 0.0000 | NA | NA | NA | 0.7581 |
| **HIV** | 0.0000 | 6.5314 | 0.0000 | 0.0000 | 20.2903 | 0.0000 | 0.0000 | 0.0000 | 0.0000 | 0.0000 | 0.0000 | 0.0000 | 0.0000 | 2.3660 | 0.0000 | 6.6534 | 2.5371 | 0.0000 | 1.7926 | 17.6482 | 14.1231 | NA | NA | 0.0000 | 0.0000 |
| **Proteinuria** | 0.5496 | 4.4860 | 0.4734 | 4.3394 | 3.5895 | 3.4874 | 2.4840 | 1.9685 | 0.0000 | 0.0000 | 0.3981 | 1.4625 | 0.0000 | 4.6384 | NA | 17.9795 | 16.9289 | 14.0714 | 0.8444 | 1.2557 | 9.5311 | NA | 0.0000 | NA | NA |
| **CKD** | 0.9298 | 2.3341 | 2.7858 | 4.1660 | 3.4954 | 3.9484 | 4.3085 | 2.6438 | 1.7616 | 2.1429 | 0.5544 | 3.4401 | 3.0295 | 3.8434 | 0.0000 | 2.7337 | 5.2341 | 3.4147 | 1.1444 | 1.1995 | 0.4129 | 0.7581 | 0.0000 | NA | NA |

Using logistic regression to obtain OR values. If the p-value is less than 0.05, it will be highlighted in red.
