# Supplementary material for: Exploring the impact of polychlorinated biphenyls on comorbidity and potential mitigation strategies
Source: Front Public Health. 2024 Oct 30;12:1474994. doi: 10.3389/fpubh.2024.1474994 (PMC11557481; doi:10.3389/fpubh.2024.1474994)
Supplement: Supplementary file 3 [file Table_3.docx]

**Supplementary Table 3 The logistic regression results for the most relevant disease pairs.**

| Disease | Most relevant disease | OR (95%CI) | P value |
| --- | --- | --- | --- |
| Asthma | CB | 5.85(4.37,7.83) | <0.0001 |
| Anemia | HIV | 6.53(2.19,15.84) | 0.0001 |
| Arthritis | Osteoporosis | 6.27(5.23,7.55) | <0.0001 |
| HF | HA | 22.26(17.20,28.79) | <0.0001 |
| HD | Angina | 35.24(27.57,45.11) | <0.0001 |
| Angina | HD | 35.24(27.57,45.12) | <0.0001 |
| HA | HD | 37.92(29.93,48.12) | <0.0001 |
| Stroke | Depression | 40.21(5.83,794.00) | 0.001 |
| Emphysema | Stroke | 6.34(4.06,9.56) | <0.0001 |
| TD | Angina | 3.60(2.76,4.66) | <0.0001 |
| CB | HPL | 5.18(2.43,13.43) | 0.0001 |
| Cancer | Depression | 13.36(2.88,69.23) | 0.0008 |
| Osteoporosis | TD | 3.85(3.09,4.77) | <0.0001 |
| Hyperuricemia | Proteinuria | 4.64(2.70,7.94) | <0.0001 |
| Depression | Stroke | 40.21(5.83,794.00) | 0.001 |
| Diabetes | Proteinuria | 17.98(10.12,32.90) | <0.0001 |
| Hypertension | Proteinuria | 16.93(7.87,44.08) | <0.0001 |
| NAFLD | Proteinuria | 14.07(2.14,54.15) | 0.0007 |
| ALD | HCV | 7.35(5.32,10.20) | <0.0001 |
| HBV | HIV | 17.65(8.54,36.26) | <0.0001 |
| HCV | HIV | 14.12(4.53,52.74) | <0.0001 |
| HPL | Diabetes | 5.21(2.65,12.27) | <0.0001 |
| HIV | HD | 20.29(5.76,55.66) | <0.0001 |
| Proteinuria | Diabetes | 17.98(10.12,32.90) | <0.0001 |
| CKD | Hypertension | 5.23(4.55,6.03) | <0.0001 |
