# Supplementary material for: Exploring the impact of polychlorinated biphenyls on comorbidity and potential mitigation strategies
Source: Front Public Health. 2024 Oct 30;12:1474994. doi: 10.3389/fpubh.2024.1474994 (PMC11557481; doi:10.3389/fpubh.2024.1474994)

**Supplementary Figure 1** Comparison of ROC curves and confusion matrices for different machine learning models before and after incorporating PCBs.

**Fig. S1a. The comparison of ROC curves and confusion matrices for the SVM model before and after incorporating PCBs.**


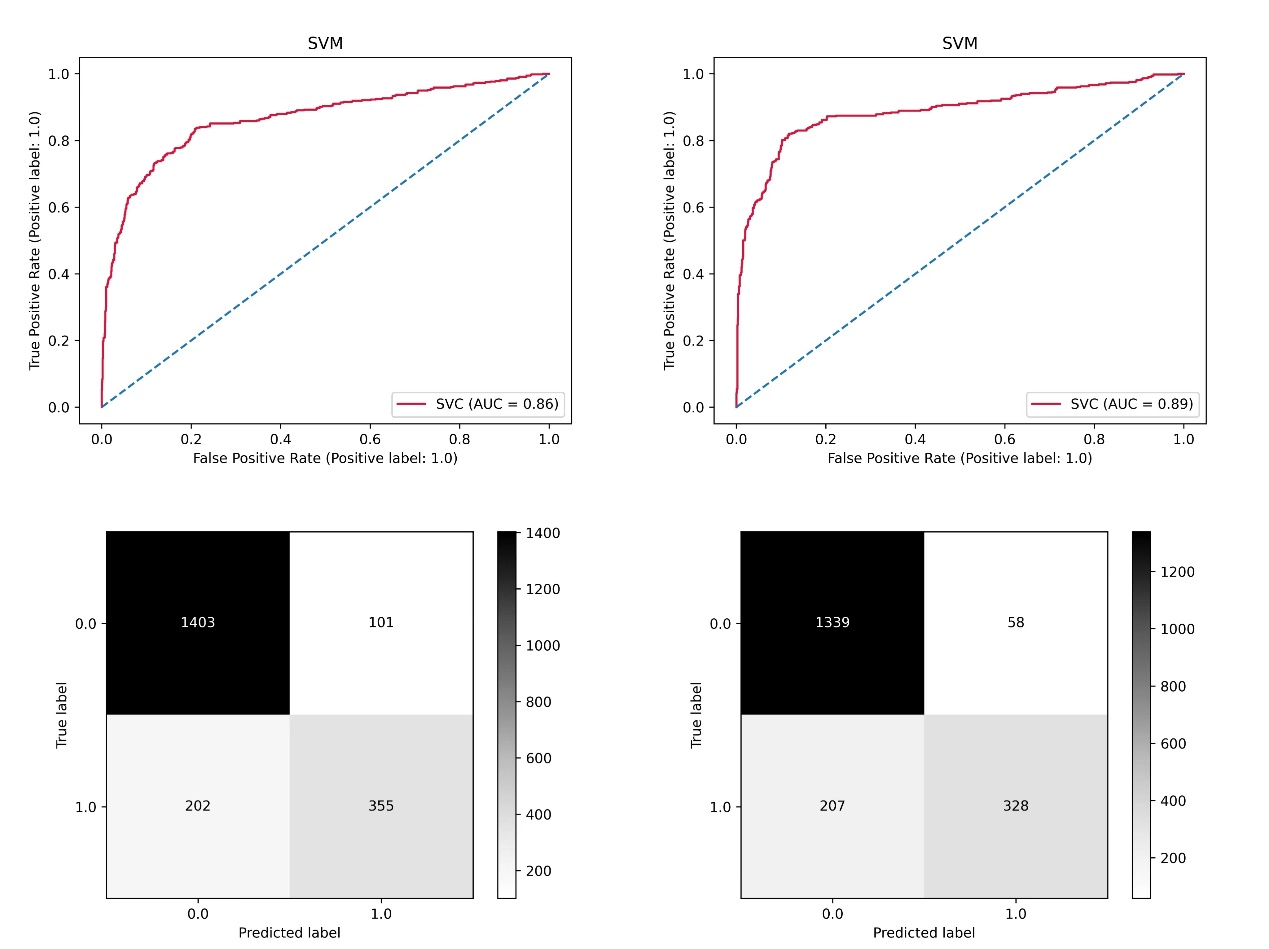


**Fig. S1b. The comparison of ROC curves and confusion matrices for the SGD model before and after incorporating PCBs.**


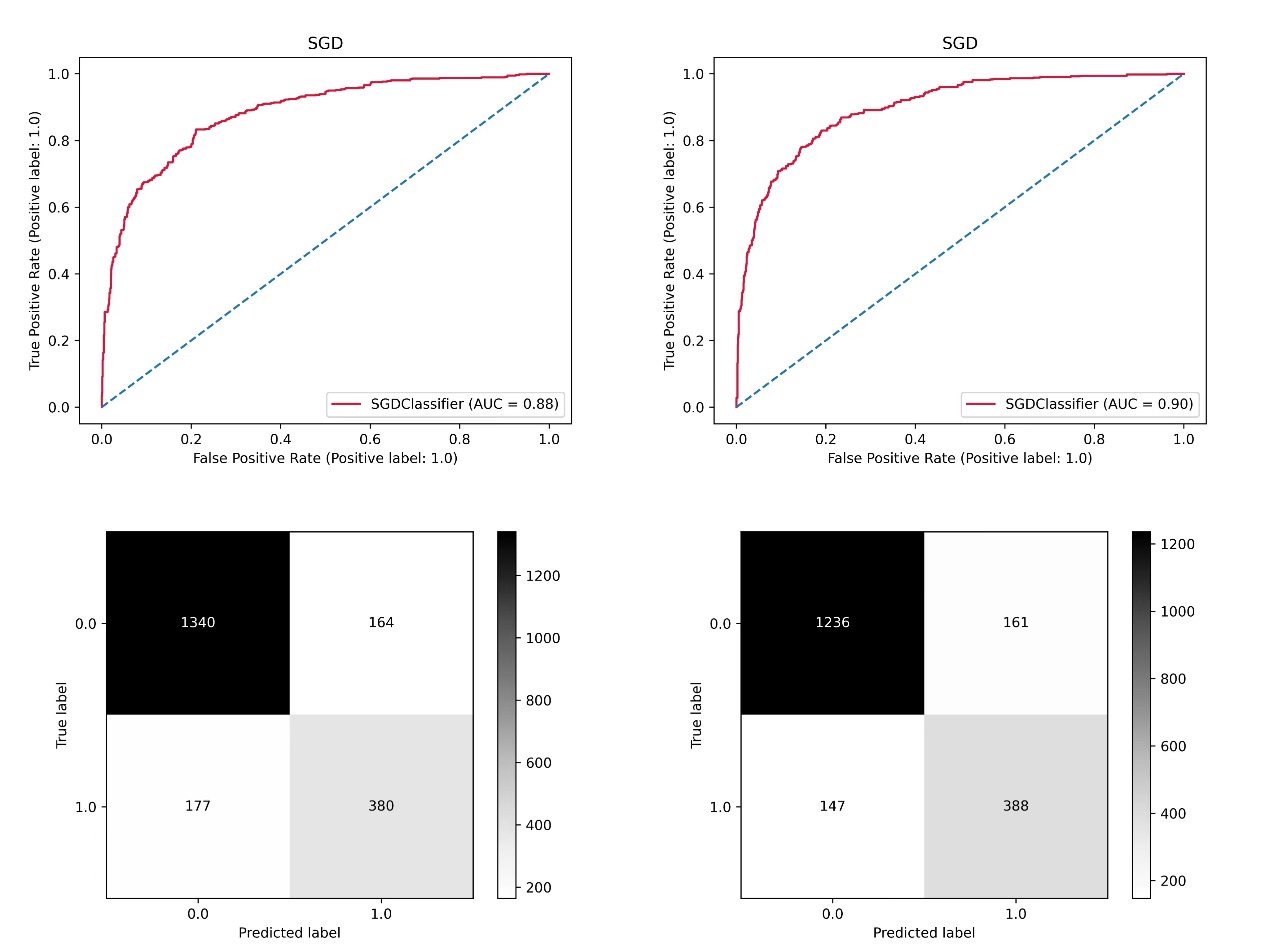


**Fig. S1c. The comparison of ROC curves and confusion matrices for the Naïve Bayes model before and after incorporating PCBs.**


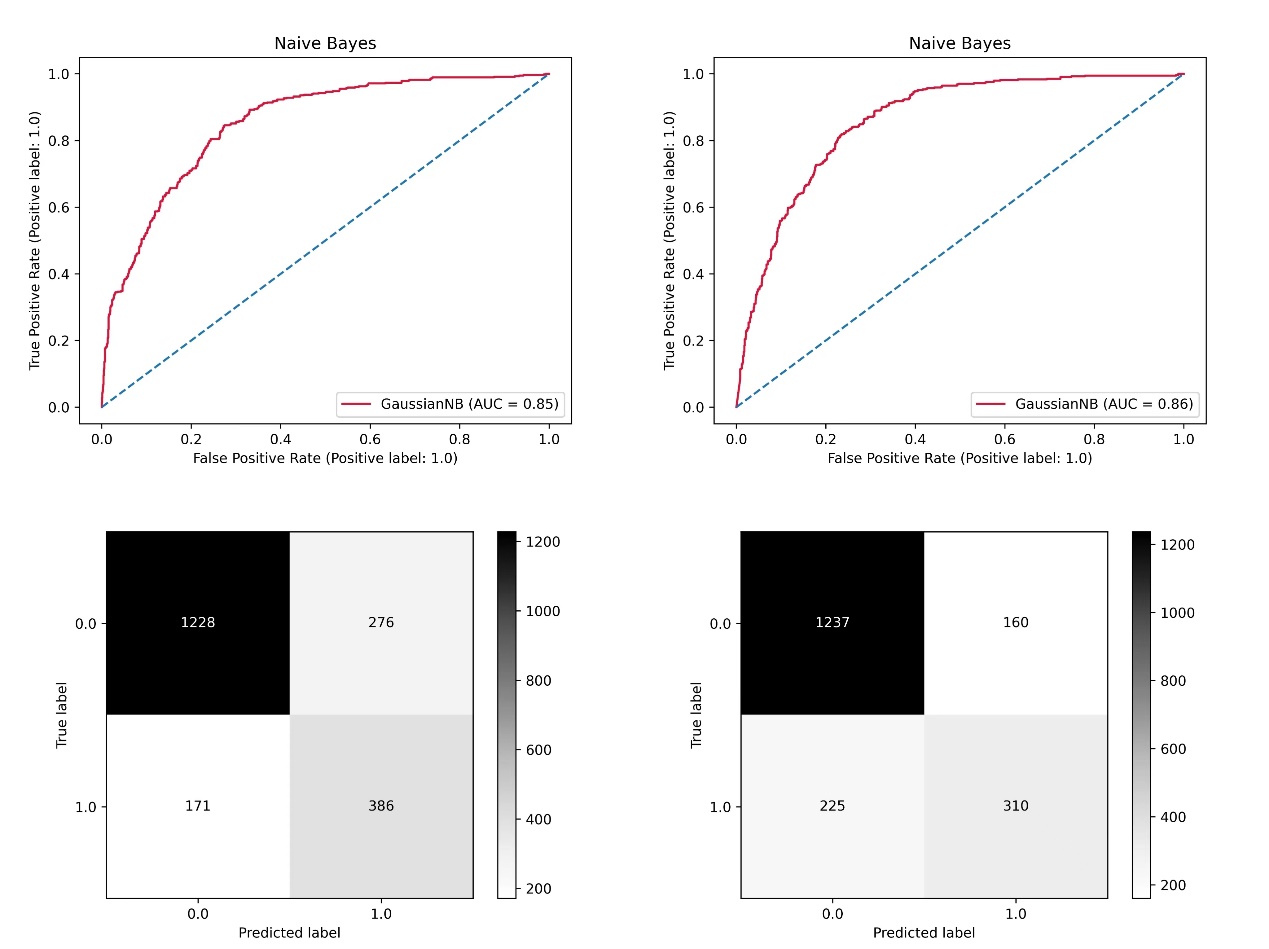


**Fig. S1d. The comparison of ROC curves and confusion matrices for the Decision Tree model before and after incorporating PCBs.**


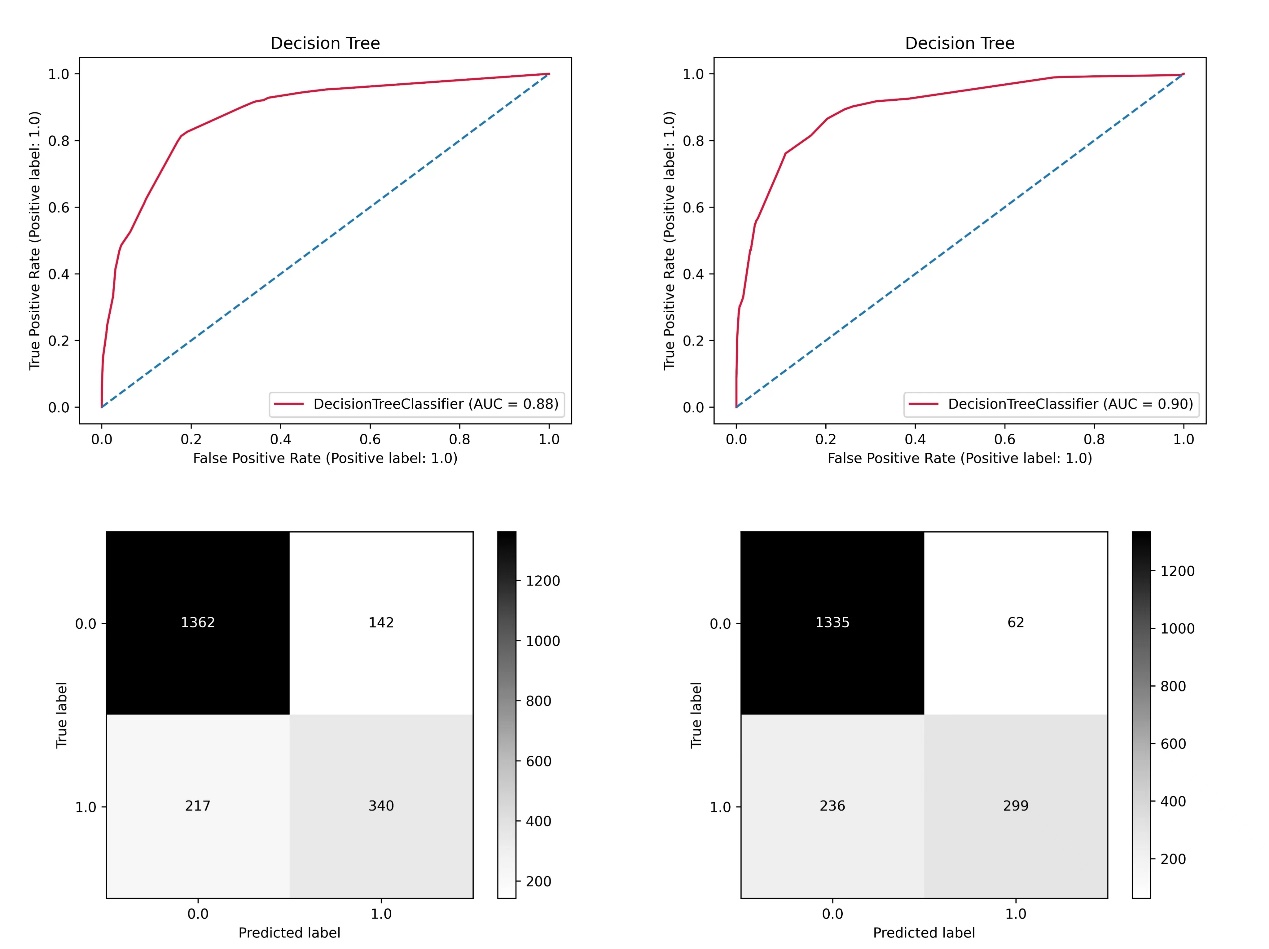


**Fig. S4e. The comparison of ROC curves and confusion matrices for the GBDT model before and after incorporating PCBs.**


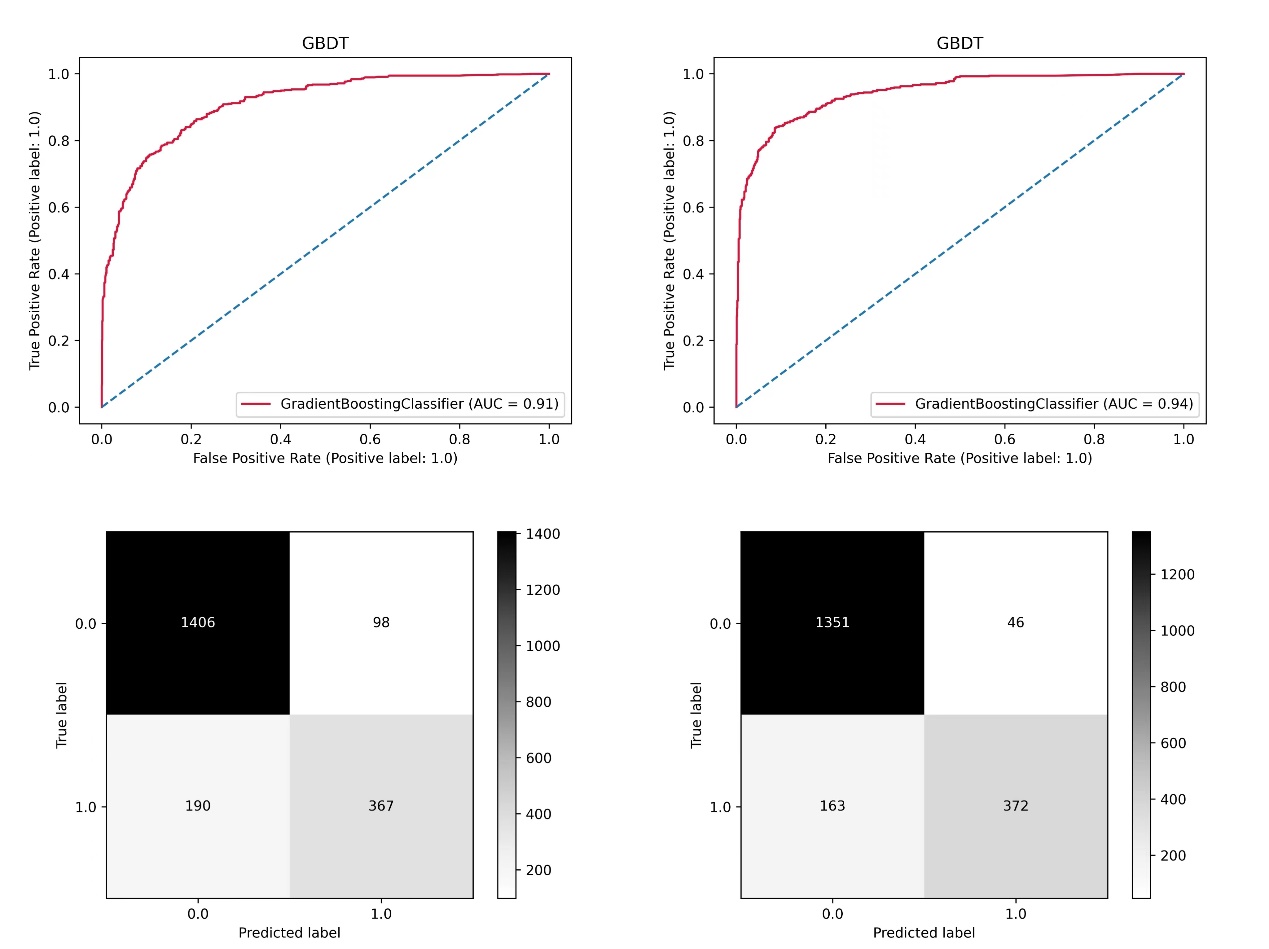


**Fig. S1f. The comparison of ROC curves and confusion matrices for the hist GBDT model before and after incorporating PCBs.**


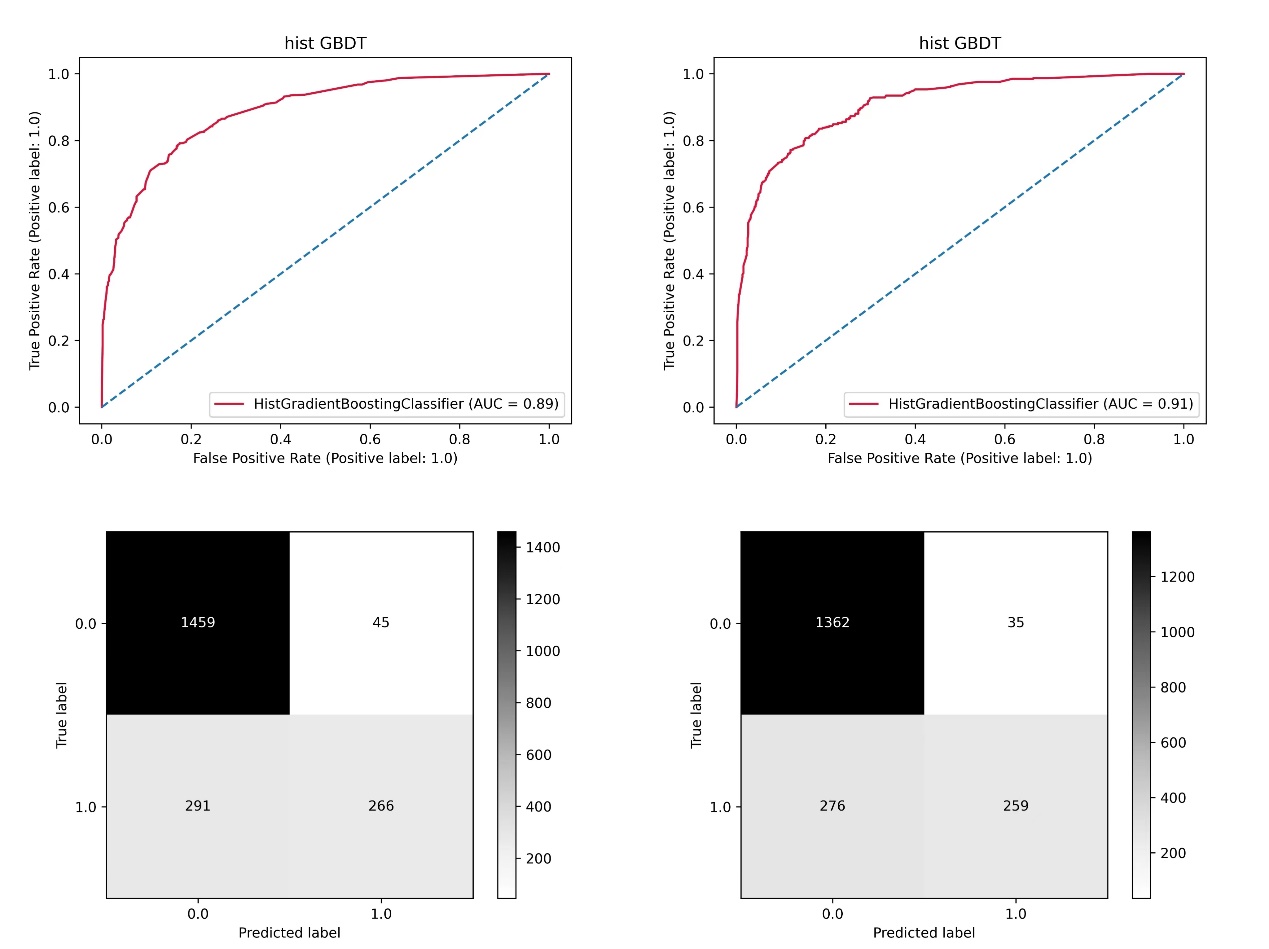


**Fig. S1g. The comparison of ROC curves and confusion matrices for the Random Forests model before and after incorporating PCBs.**


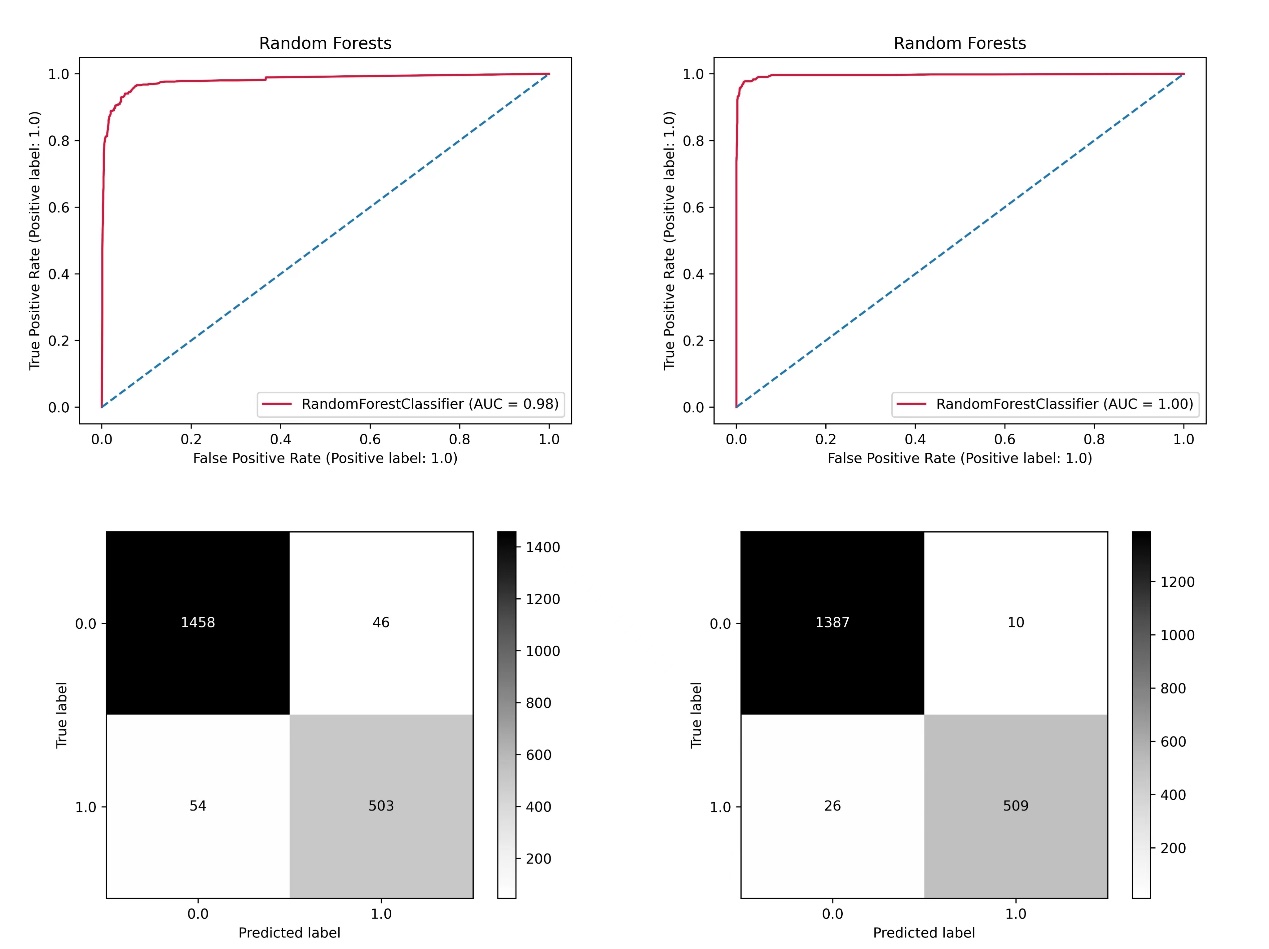


**Fig. S1h. The comparison of ROC curves and confusion matrices for the Bagging model before and after incorporating PCBs.**


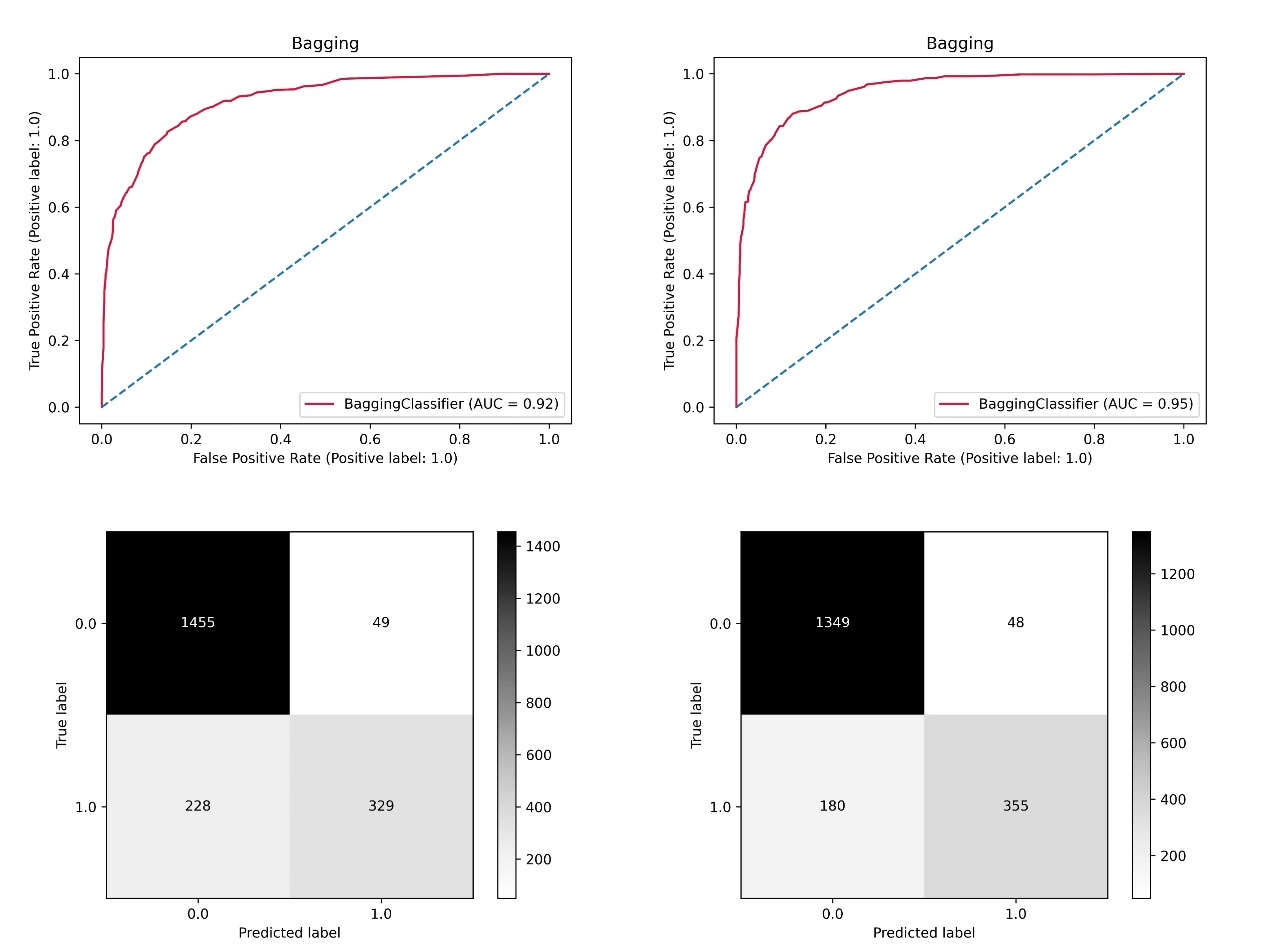


**Fig. S1i. The comparison of ROC curves and confusion matrices for the Neural Network model before and after incorporating PCBs.**


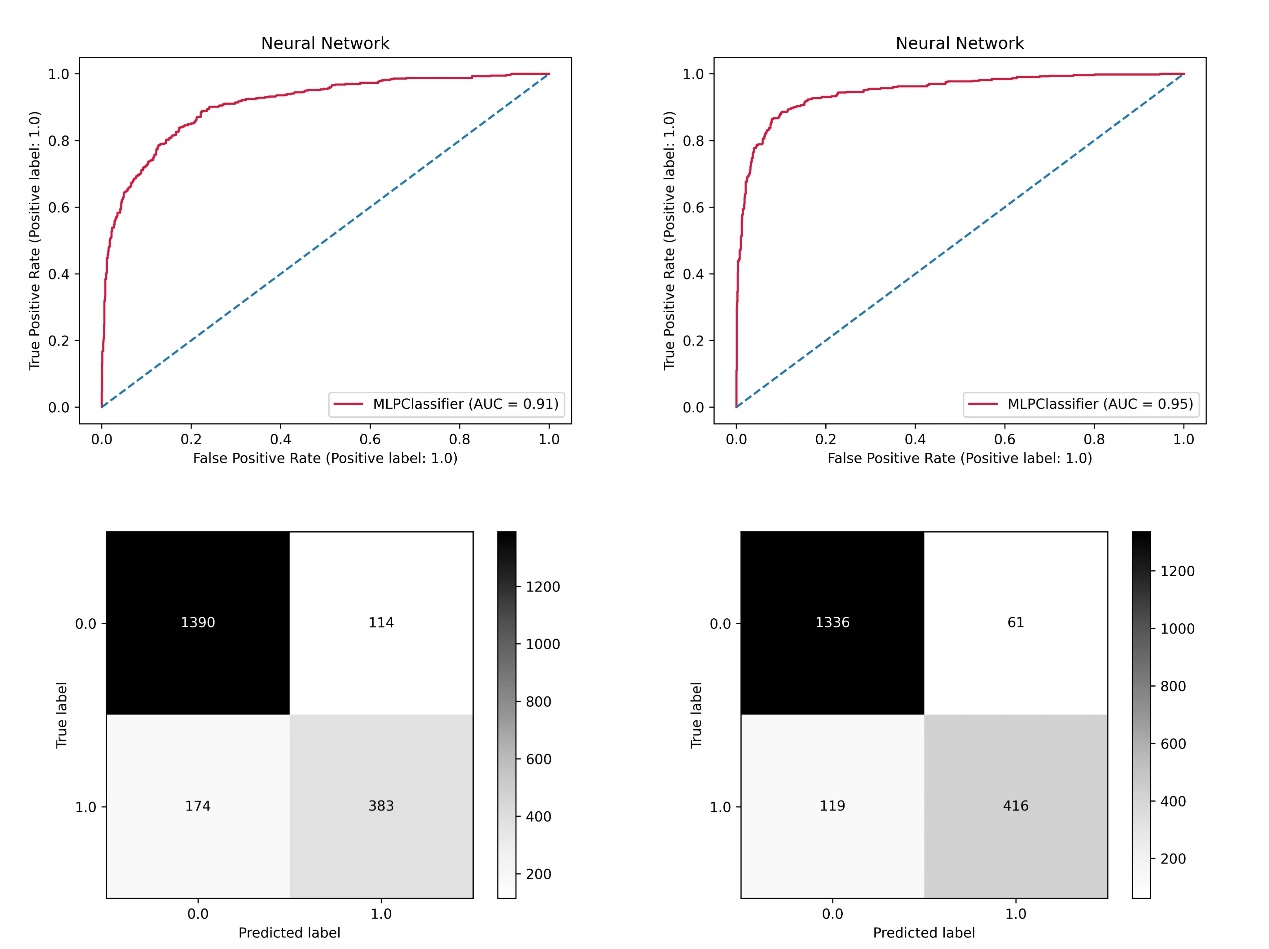


**Fig. S1j. The comparison of ROC curves and confusion matrices for the Voting model before and after incorporating PCBs.**


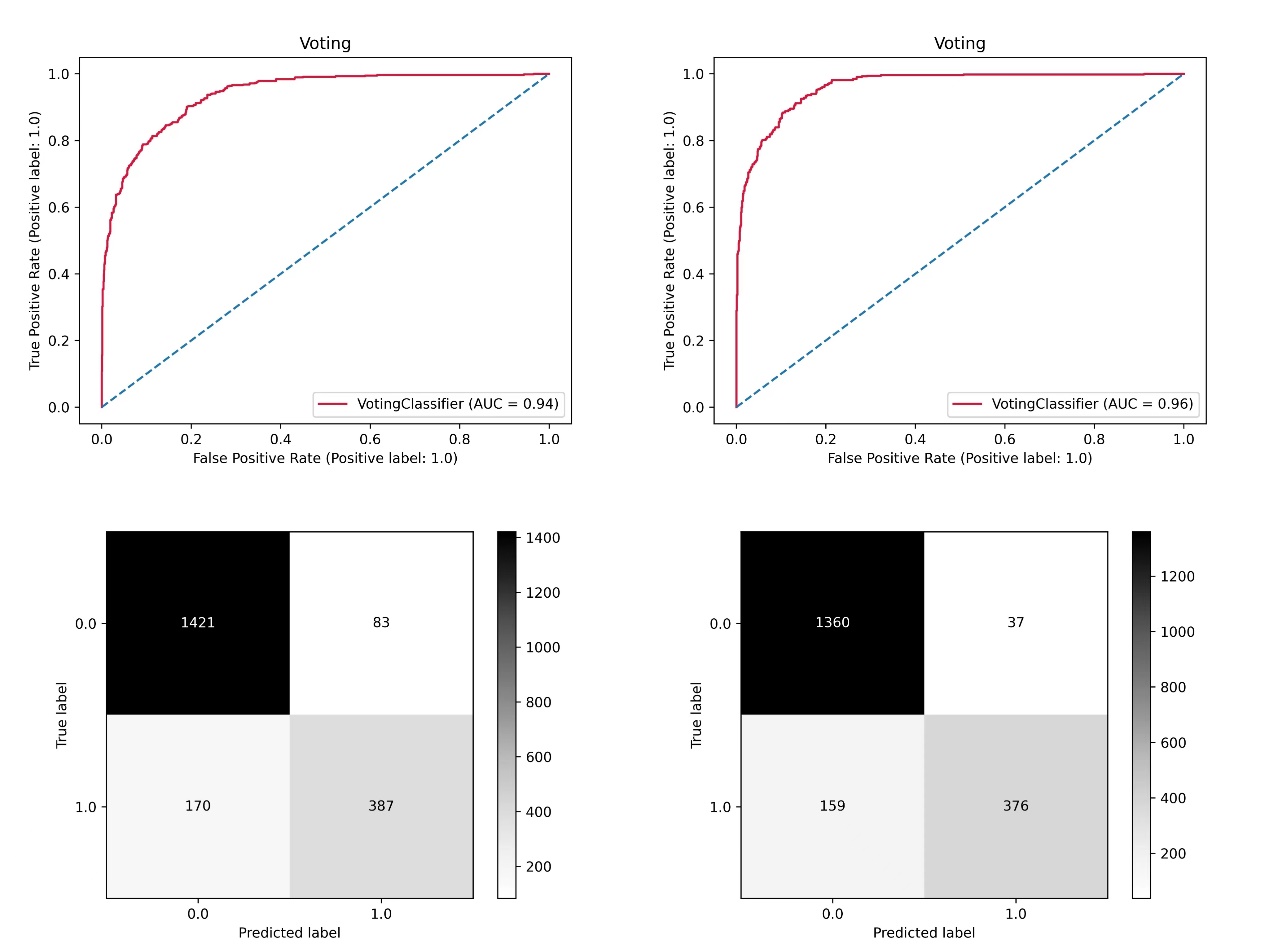

Supplement: Supplementary file 4 [file Data_Sheet_1.docx]
