# Supplementary material for: Exploring the impact of polychlorinated biphenyls on comorbidity and potential mitigation strategies
Source: Front Public Health. 2024 Oct 30;12:1474994. doi: 10.3389/fpubh.2024.1474994 (PMC11557481; doi:10.3389/fpubh.2024.1474994)
Supplement: Supplementary file 5 [file Data_Sheet_2.docx]

**Supplementary Figure 2 Multivariable Cox regression forest plot of PCBs and mortality risk.**

**Figure S2a. Multivariable Cox regression forest plot of PCB74 and mortality risk.**


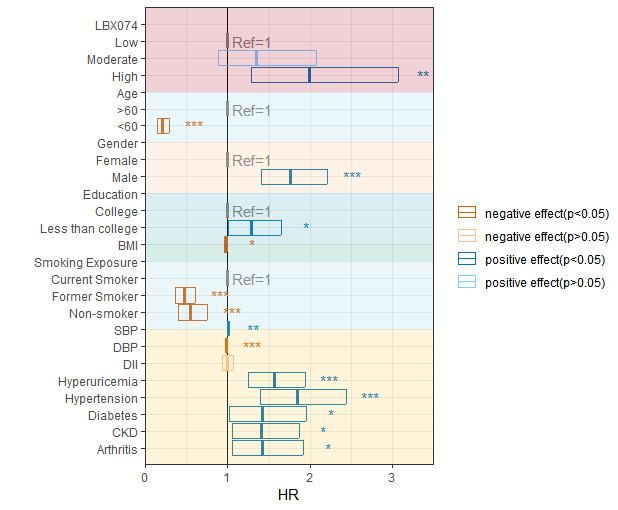


Based on the different exposure levels of PCB74 in participants, they were divided into three groups—"Low", "Moderate," and "High"—according to the terciles of PCB74 concentration, with "Low" serving as the control group. Hazard Ratios (HR) less than 1 are represented in red, while HR greater than 1 are represented in blue. After adjusting for factors such as age, gender, education level, BMI, smoking exposure, SII, and exposure to five diseases, the impact of PCB74 on mortality risk was examined.

* 0.001 < P ≤ 0.05

** 0.0001 < P ≤ 0.01

*** P ≤ 0.0001

**Figure S2b. Multivariable Cox regression forest plot of PCB170 and mortality risk.**


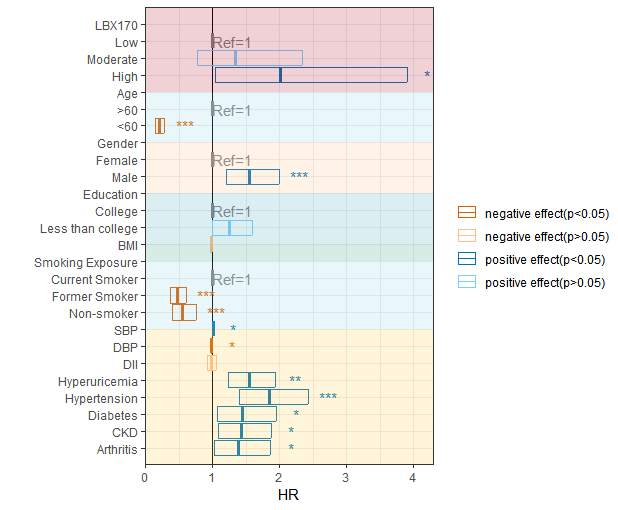


Based on the different exposure levels of PCB170 in participants, they were divided into two groups—"Low" and "High"—according to the terciles of PCB170 concentration, with "Low" serving as the control group. Hazard Ratios (HR) less than 1 are represented in red, while HR greater than 1 are represented in blue. After adjusting for factors such as age, gender, education level, BMI, smoking exposure, SII, and exposure to five diseases, the impact of PCB170 on mortality risk was examined.

* 0.001 < P ≤ 0.05

** 0.0001 < P ≤ 0.01

*** P ≤ 0.0001

**Figure S2c. Multivariable Cox regression forest plot of PCB178 and mortality risk.**


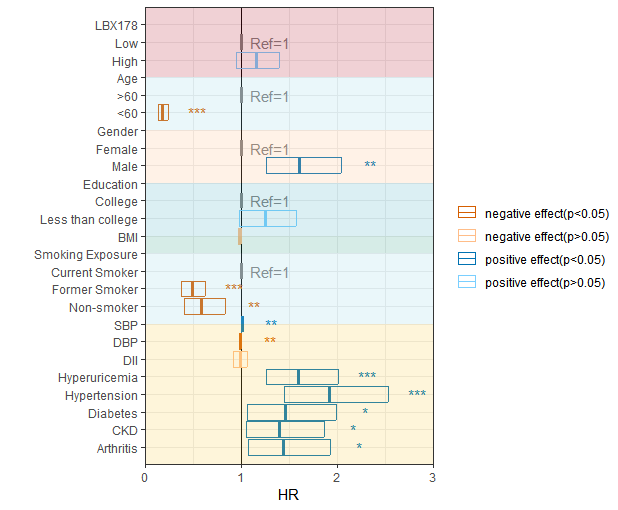


Based on the different exposure levels of PCB178 in participants, they were divided into three groups—"Low", "Moderate," and "High"—according to the terciles of PCB178 concentration, with "Low" serving as the control group. Hazard Ratios (HR) less than 1 are represented in red, while HR greater than 1 are represented in blue. After adjusting for factors such as age, gender, education level, BMI, smoking exposure, SII, and exposure to five diseases, the impact of PCB178 on mortality risk was examined.

* 0.001 < P ≤ 0.05

** 0.0001 < P ≤ 0.01

*** P ≤ 0.0001

**Figure S2d. Multivariable Cox regression forest plot of PCB180 and mortality risk.**


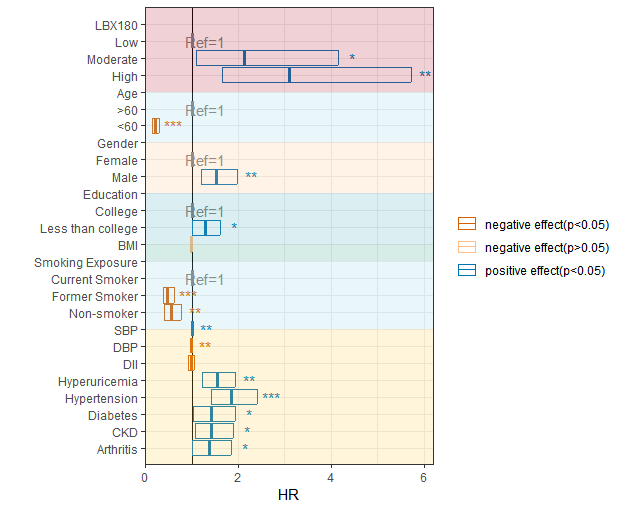


Based on the different exposure levels of PCB180 in participants, they were divided into three groups—"Low", "Moderate," and "High"—according to the terciles of PCB180 concentration, with "Low" serving as the control group. Hazard Ratios (HR) less than 1 are represented in red, while HR greater than 1 are represented in blue. After adjusting for factors such as age, gender, education level, BMI, smoking exposure, SII, and exposure to five diseases, the impact of PCB180 on mortality risk was examined.

* 0.001 < P ≤ 0.05

** 0.0001 < P ≤ 0.01

*** P ≤ 0.0001

**Figure S2e. Multivariable Cox regression forest plot of PCB156 and mortality risk.**


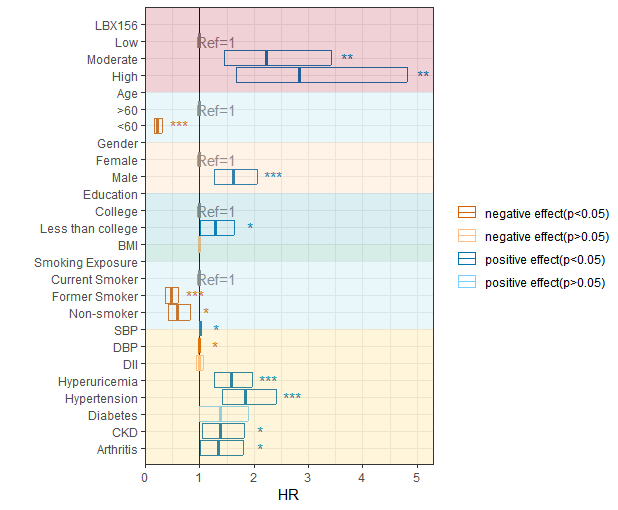


Based on the different exposure levels of PCB156 in participants, they were divided into three groups—"Low", "Moderate," and "High"—according to the terciles of PCB156 concentration, with "Low" serving as the control group. Hazard Ratios (HR) less than 1 are represented in red, while HR greater than 1 are represented in blue. After adjusting for factors such as age, gender, education level, BMI, smoking exposure, SII, and exposure to five diseases, the impact of PCB156 on mortality risk was examined.

* 0.001 < P ≤ 0.05

** 0.0001 < P ≤ 0.01

*** P ≤ 0.0001

**Figure S2f. Multivariable Cox regression forest plot of PCB157 and mortality risk.**


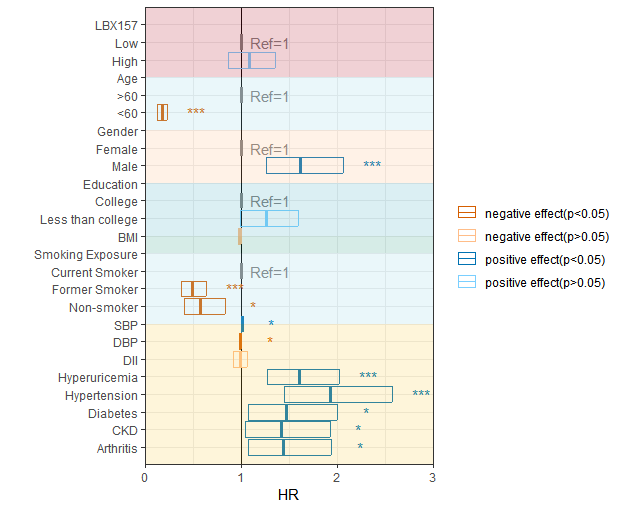


Based on the different exposure levels of PCB157 in participants, they were divided into two groups—"Low" and "High"—according to the terciles of PCB157 concentration, with "Low" serving as the control group. Hazard Ratios (HR) less than 1 are represented in red, while HR greater than 1 are represented in blue. After adjusting for factors such as age, gender, education level, BMI, smoking exposure, SII, and exposure to five diseases, the impact of PCB157 on mortality risk was examined.

* 0.001 < P ≤ 0.05

** 0.0001 < P ≤ 0.01

*** P ≤ 0.0001

**Figure S2g. Multivariable Cox regression forest plot of PCB146 and mortality risk.**


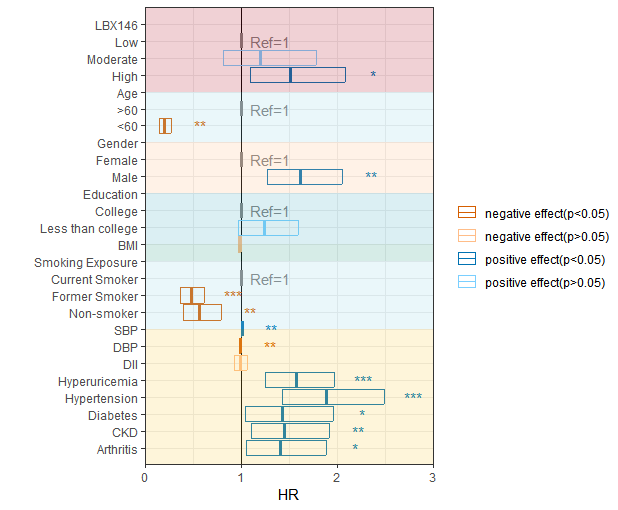


Based on the different exposure levels of PCB146 in participants, they were divided into three groups—"Low", "Moderate," and "High"—according to the terciles of PCB146 concentration, with "Low" serving as the control group. Hazard Ratios (HR) less than 1 are represented in red, while HR greater than 1 are represented in blue. After adjusting for factors such as age, gender, education level, BMI, smoking exposure, SII, and exposure to five diseases, the impact of PCB146 on mortality risk was examined.

* 0.001 < P ≤ 0.05

** 0.0001 < P ≤ 0.01

*** P ≤ 0.0001
